# Supplementary figures and images for: LSM12-EPAC1 defines a neuroprotective pathway that sustains the nucleocytoplasmic RAN gradient
Source: PLoS Biol. 2020 Dec 23;18(12):e3001002. doi: 10.1371/journal.pbio.3001002 (PMC7757817; doi:10.1371/journal.pbio.3001002)

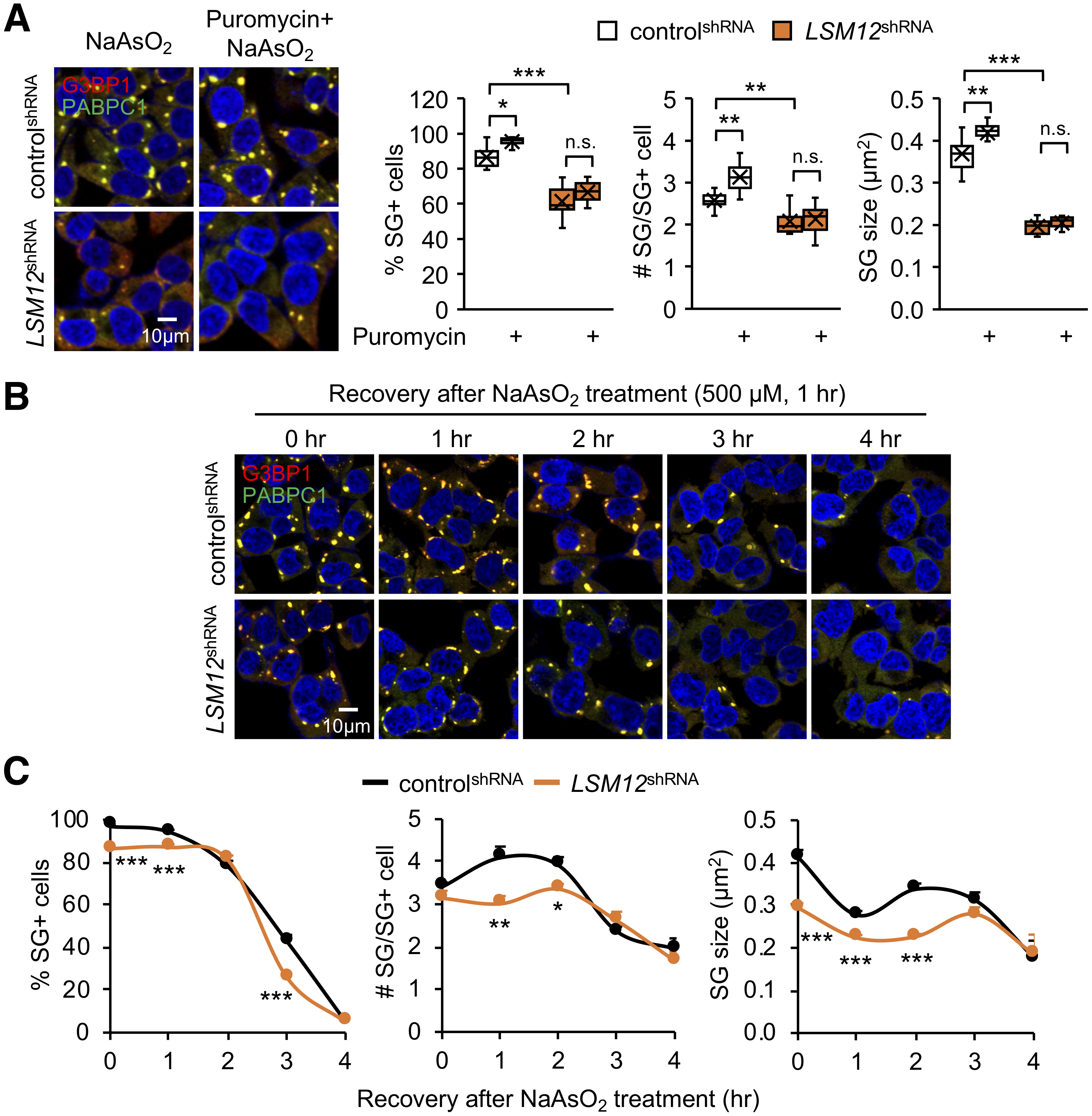

Supplement: S1 Fig — (A) Puromycin treatment enhances arsenite-induced SG formation in controlshRNA cells, but not in LSM12shRNA cells. ControlshRNA and LSM12shRNA cells were preincubated with puromycin (5 μg/ml) or PBS (vehicle control) for 15 minutes before the induction of chronic oxidative stress (50-μM NaAsO2, 2 hours). Cells were co-stained with anti-G3BP1 antibody (red), anti-PABPC1 antibody (green), and Hoechst 33258 (blue) to visualize SGs and the nucleus, respectively. The percentage of SG-positive cells, the number of SGs per cell, and the size of SGs were quantified using ImageJ software and averaged (n = 10 confocal images obtained from 3 independent experiments; n = 713–939 cells). Error bars indicate SEM. n.s., not significant; *P < 0.05, **P < 0.01, ***P < 0.001, as determined by 2-way ANOVA with Tukey post hoc test. (B) LSM12 depletion altered the kinetics of SG disassembly during the recovery from acute oxidative stress. ControlshRNA and LSM12shRNA cells were incubated with 500-μM NaAsO2 for 1 hour and then washed with fresh media. Cells were fixed at the indicated time after the removal of NaAsO2 and co-stained with anti-G3BP1 antibody (red), anti-PABPC1 antibody (green), and Hoechst 33258 (blue). (C) The kinetics of SG disassembly were quantified similarly as above. Data represent means ± SEM (n = 8–10 confocal images obtained from 3 independent experiments; n = 388–740 cells). *P < 0.05, **P < 0.01, ***P < 0.001, as determined by Student t test. All underlying numerical values are available in S1 Data. ANOVA, analysis of variance; LSM12, like-Sm protein 12; SG, stress granule; SEM, standard error of the mean. (TIFF) [file pbio.3001002.s001.tiff]

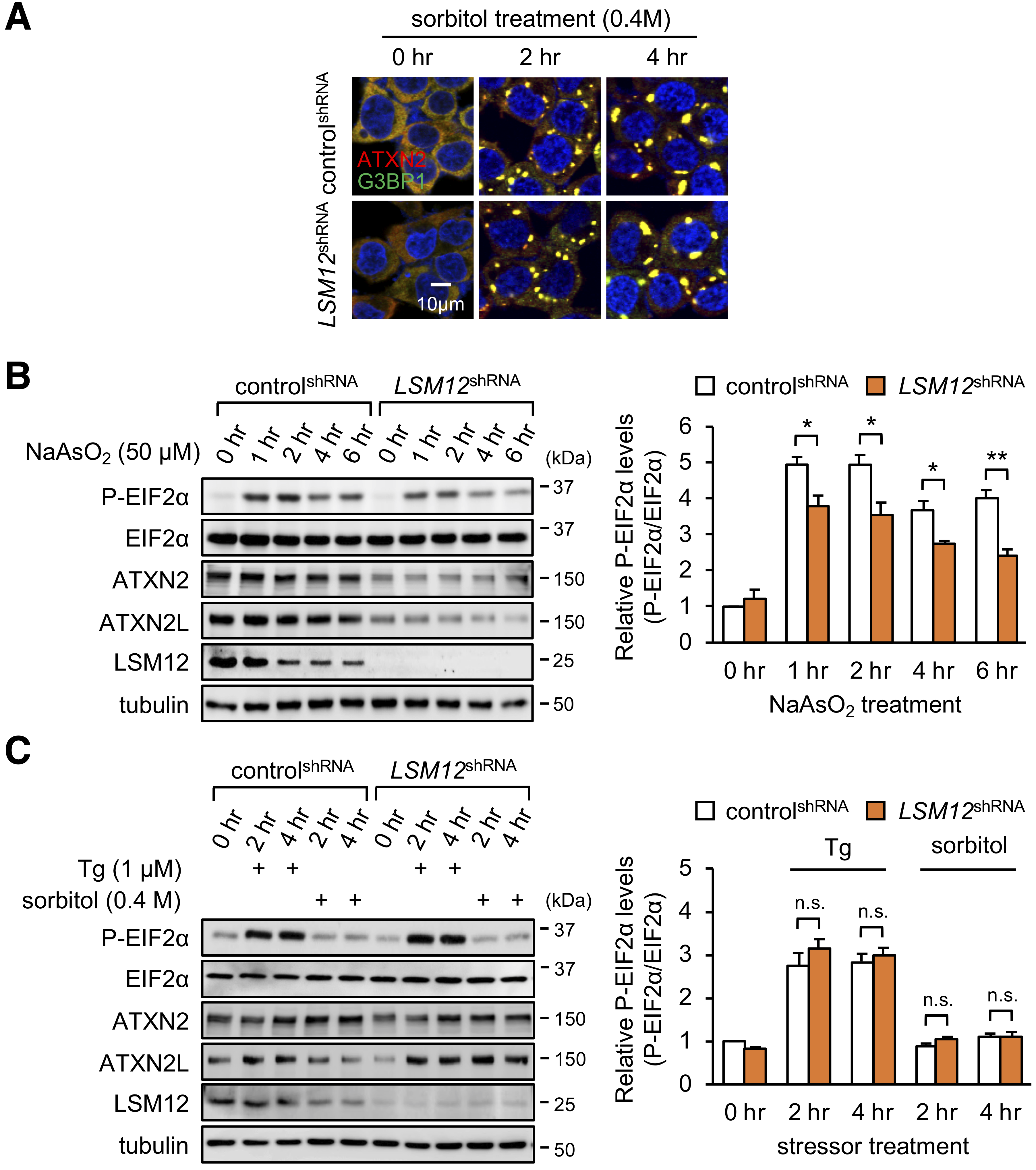

Supplement: S2 Fig — (A) LSM12 depletion does not suppress the SG assembly under sorbitol-induced osmotic stress. ControlshRNA and LSM12shRNA cells were incubated with 0.4-M sorbitol for the indicated time before co-staining with anti-ATXN2 antibody (red), anti-G3BP1 antibody (green), and Hoechst 33258 (blue). (B, C) LSM12 depletion decreases the arsenite-induced phosphorylation of EIF2α. ControlshRNA and LSM12shRNA cells were incubated with 50-μM NaAsO2, 1-μM thapsigargin (Tg), or 0.4-M sorbitol for the indicated time before harvest. Whole-cell lysates were immunoblotted with specific antibodies (left). Relative levels of EIF2α phosphorylation were calculated by normalizing the ratio of phospho-EIF2α to EIF2α protein levels per condition to that in control cells with no chemical treatment. Data represent means ± SEM (n = 3). n.s., not significant; *P < 0.05, **P < 0.01, as determined by Student t test. All underlying numerical values are available in S1 Data. ATXN2, ataxin-2; EIF2α, eukaryotic translation initiation factor 2 subunit α; LSM12, like-Sm protein 12; SEM, standard error of the mean; SG, stress granule. (TIFF) [file pbio.3001002.s002.tiff]

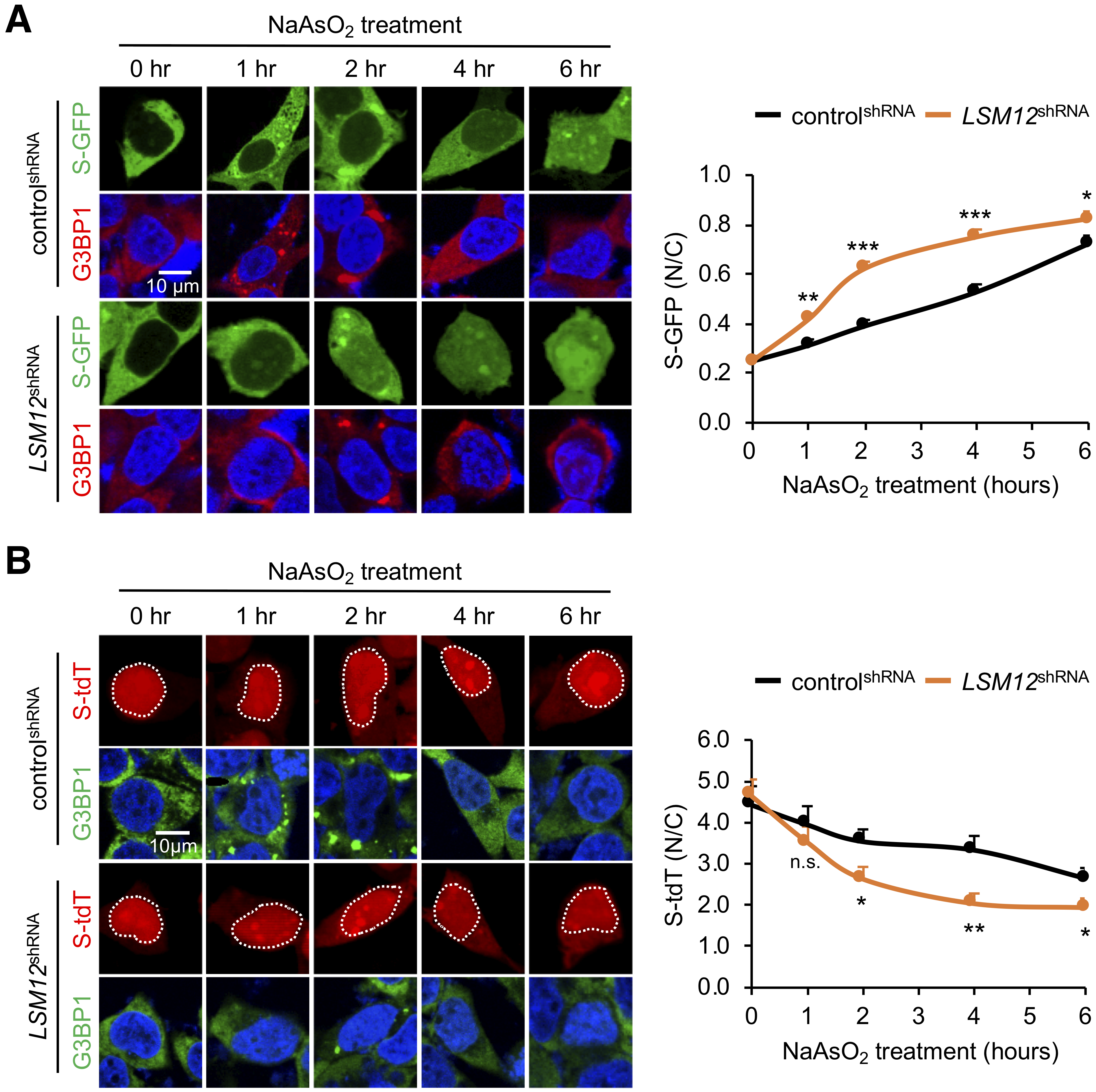

Supplement: S3 Fig — (A) LSM12 depletion facilitates the nuclear mislocalization of S-GFP under oxidative stress conditions. ControlshRNA and LSM12shRNA cells were transfected with an S-GFP expression vector and then treated with 50-μM NaAsO2 for the indicated time before co-staining with anti-G3BP1 antibody (red) and Hoechst 33258 (blue). NCT of S-GFP reporter proteins was quantified as in Fig 2B. Data represent means ± SEM (n = 123–127 cells from 3 independent experiments). *P < 0.05, **P < 0.01, ***P < 0.001 to controlshRNA cells at a given time point, as determined by Student t test. (B) LSM12 depletion facilitates the cytoplasmic mislocalization of S-tdT under oxidative stress conditions. Data represent means ± SEM (n = 103–118 cells from 3 independent experiments). n.s., not significant; *P < 0.05, **P < 0.01 to controlshRNA cells at a given time point, as determined by Student t test. All underlying numerical values are available in S1 Data. GFP, green fluorescent protein; LSM12, like-Sm protein 12; NCT, nucleocytoplasmic transport; S-tdT, S-tdTomato; SEM, standard error of the mean. (TIFF) [file pbio.3001002.s003.tiff]

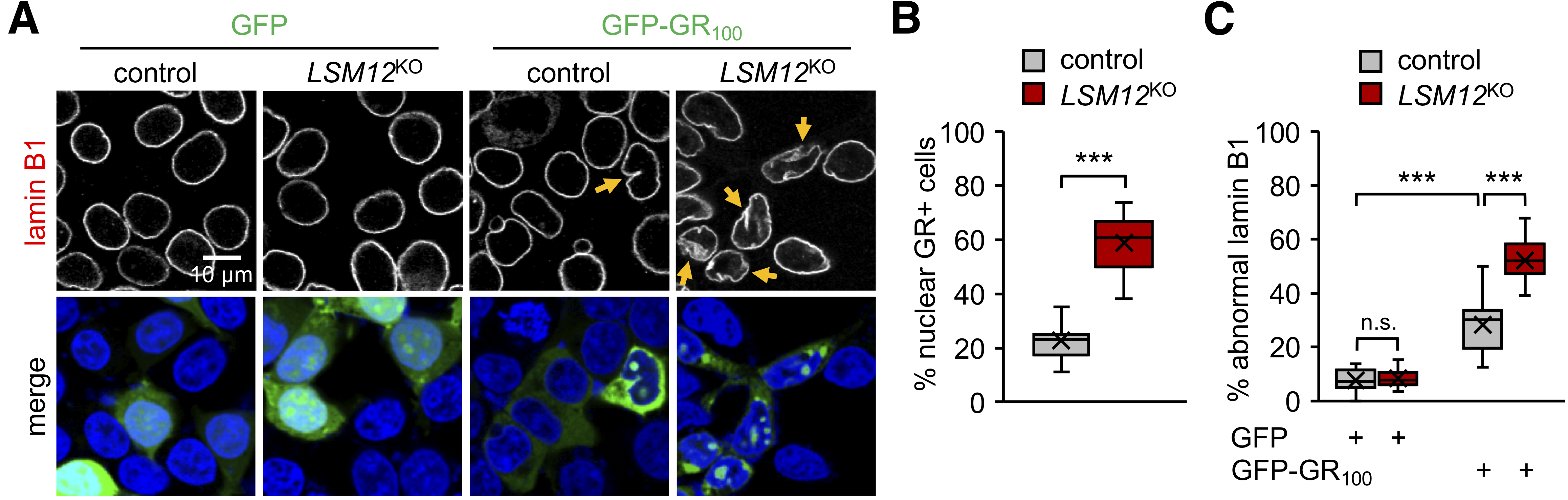

Supplement: S4 Fig — (A) LSM12 deletion exacerbates the poly(GR)-induced invaginations of the nuclear envelope. Control and LSM12KO cells were transfected with a GFP-GR100 expression vector and then co-stained with anti-lamin B1 antibody (red) and Hoechst 33258 (blue) 48 hours after transfection. Yellow arrows indicate GFP-GR100–positive cells with severe nuclear lamina disruption. (B) The assembly of nuclear poly(GR) granules was quantified as in Fig 1. Data represent mean ± SEM (n = 15–17 confocal images obtained from 3 independent experiments; n = 403–418 GFP-GR100–positive cells). ***P < 0.001, as determined by Student t test. (C) The abnormal morphology of the nuclear lamina was quantified as in Fig 3E. Data represent means ± SEM (n = 18–19 confocal images obtained from 3 independent experiments; n = 366–413 GFP–or GFP-GR100–positive cells). n.s., not significant; ***P < 0.001, as determined by 2-way ANOVA with Tukey post hoc test. All underlying numerical values are available in S1 Data. ANOVA, analysis of variance; GFP, green fluorescent protein; LSM12, like-Sm protein 12; SEM, standard error of the mean. (TIFF) [file pbio.3001002.s004.tiff]

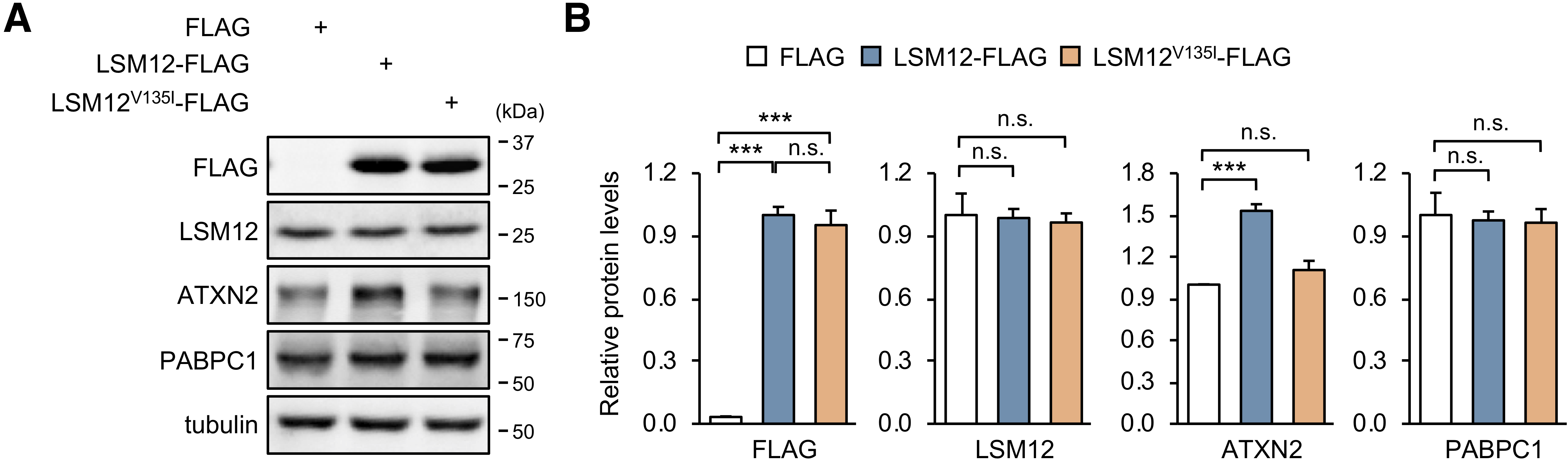

Supplement: S5 Fig — (A) SH-SY5Y cells were transfected with an expression vector for FLAG, LSM12-FLAG, or LSM12V135I-FLAG. Total cell extracts were prepared 48 hours after transfection and immunoblotted with anti-FLAG, anti-LSM12, anti-ATXN2, anti-PABPC1, and anti-tubulin (loading control) antibodies. Overexpression of wild-type LSM12, but not LSM12V135I, increased the relative levels of endogenous ATXN2 protein, consistent with low levels of endogenous ATXN2 protein in LSM12-depleted cells (Fig 1C). (B) The abundance of each protein was quantified as in Fig 1C. Data represent means ± SEM (n = 4). n.s., not significant; ***P < 0.001, as determined by 1-way ANOVA with Dunnett post hoc test. All underlying numerical values are available in S1 Data. ANOVA, analysis of variance; ATXN2, ataxin-2; LSM12, like-Sm protein 12; SEM, standard error of the mean. (TIFF) [file pbio.3001002.s005.tiff]

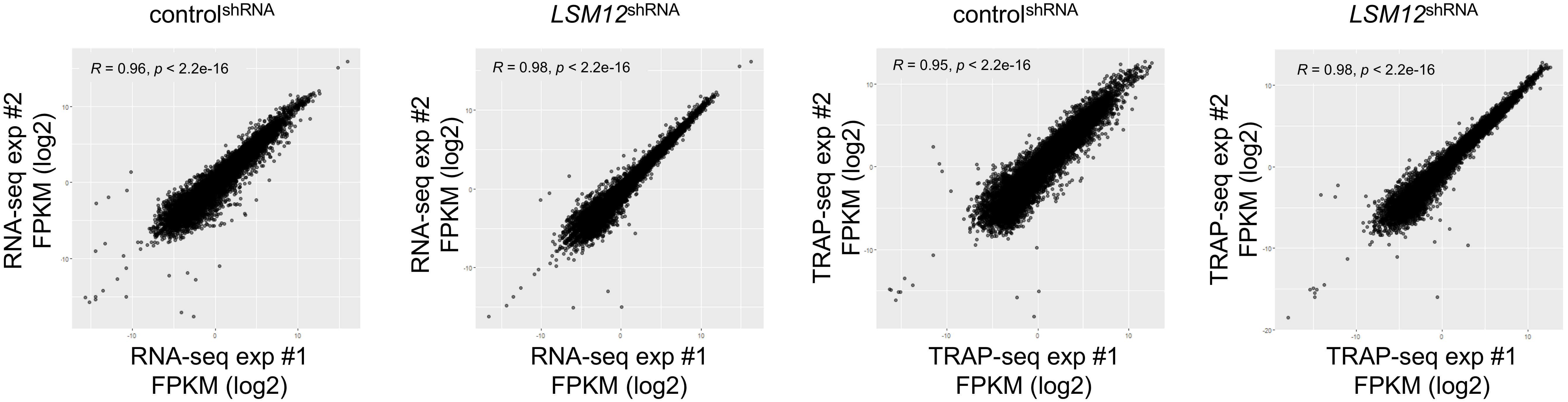

Supplement: S6 Fig — The scatter plots of RNA-seq and TRAP-seq analyses were obtained from 2 biological replicates of controlshRNA and LSM12shRNA cells. Pearson correlation and P values are indicated in each plot. All underlying numerical values are available in S1 Data. LSM12, like-Sm protein 12; RNA-seq, RNA sequencing; TRAP-seq, translating ribosome affinity purification sequencing. (TIFF) [file pbio.3001002.s006.tiff]

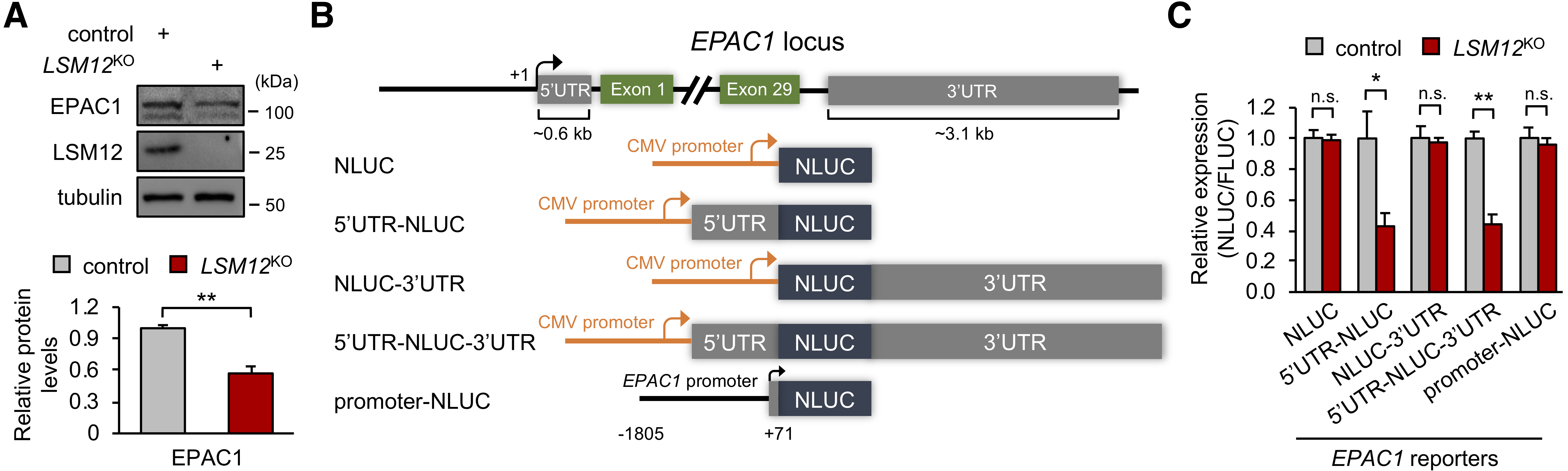

Supplement: S7 Fig — (A) LSM12-deleted (LSM12KO) cells express low levels of EPAC1 protein. The abundance of each protein was quantified as in Fig 1C. Data represent means ± SEM (n = 3). **P < 0.01, as determined by Student t test. (B) A schematic representation of the EPAC1 locus and EPAC1 reporter constructs. Transcription of control and EPAC1 UTR reporters was driven by heterologous CMV promoter. A promoter region in the EPAC1 locus (from −1,805 to +71 in relative to the transcription start site +1) was subcloned upstream of the NLUC-coding sequence to measure the EPAC1 promoter activity by the NLUC activity. (C) LSM12 deletion posttranscriptionally decreases EPAC1 expression via the 5′ UTR. Control and LSM12KO cells were co-transfected with each EPAC1 reporter and a FLUC expression vector (normalizing control). Luciferase reporter assays were performed as in Fig 5D. Data represent means ± SEM (n = 3). n.s., not significant; *P < 0.05, **P < 0.01 as determined by Student t test. All underlying numerical values are available in S1 Data. CMV, cytomegalovirus; EPAC1, exchange protein directly activated by cyclic AMP 1; FLUC, firefly luciferase; LSM12, like-Sm protein 12; NLUC, Nano-luciferase; SEM, standard error of the mean; UTR, untranslated region. (TIFF) [file pbio.3001002.s007.tiff]

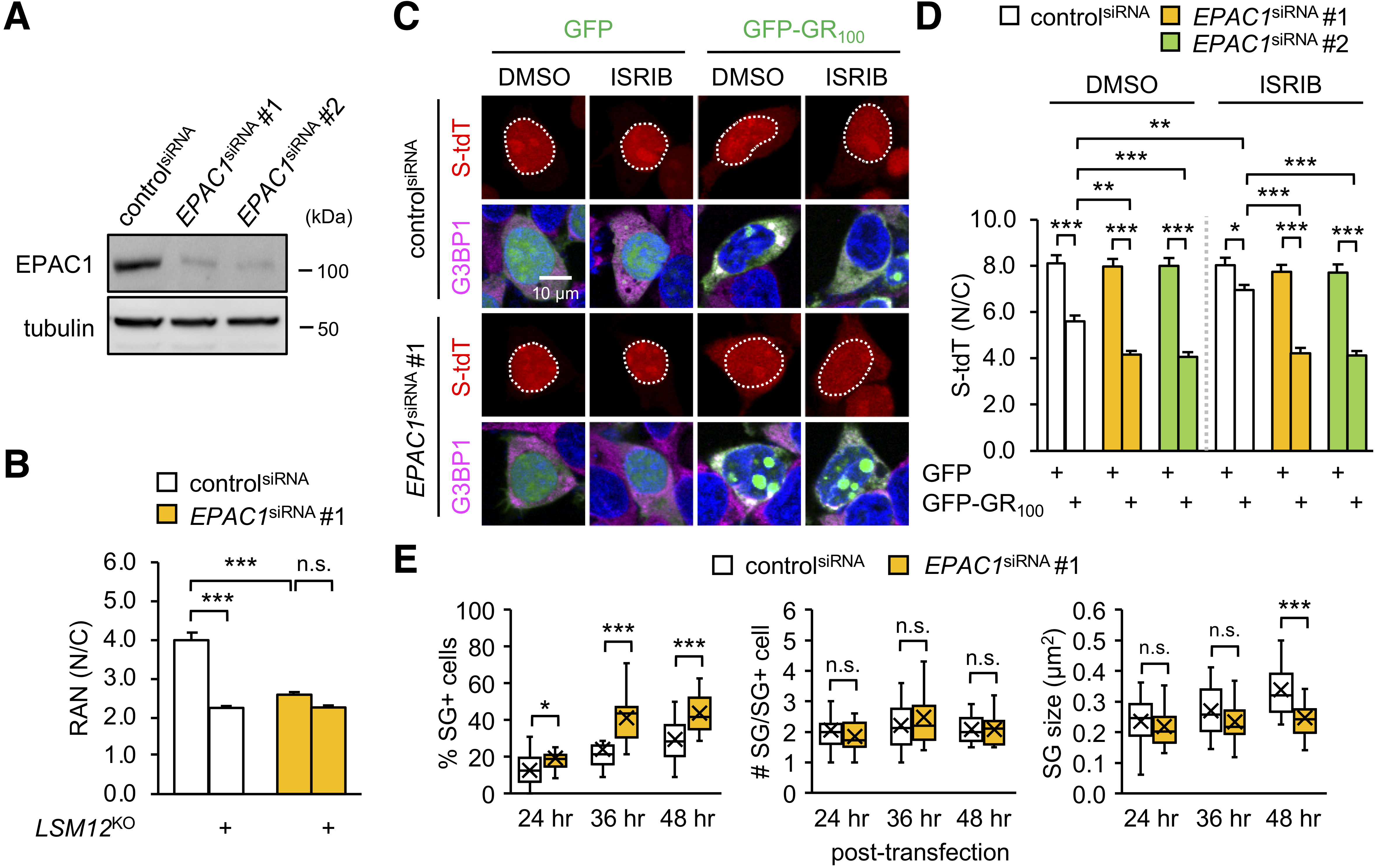

Supplement: S8 Fig — (A) SH-SY5Y cells were transfected with control or 2 independent EPAC1 siRNAs. Total cell extracts were prepared 72 hours after transfection and immunoblotted with anti-EPAC1 or anti-tubulin (loading control) antibodies. (B) LSM12 deletion and EPAC1 depletion nonadditively disrupt the RAN gradient. Control and LSM12KO cells were transfected with controlsiRNA or EPAC1siRNA. The nucleocytoplasmic RAN gradient was quantified as in Fig 2D. Data represent means ± SEM (n = 62–67 cells from 3 independent experiments). n.s., not significant; ***P < 0.001, as determined by 2-way ANOVA with Tukey post hoc test. (C, D) EPAC1 depletion exacerbates the poly(GR)-induced disruption of NCT in an ISRIB-insensitive manner. SH-SY5Y cells were co-transfected with siRNA and expression vectors for S-tdT and GFP-GR100, as in Fig 6A. Where indicated, transfected cells were incubated with 2-μM ISRIB or DMSO (vehicle control) for 5 hours before co-staining with anti-G3BP1 antibody (magenta) and Hoechst 33258 (blue) at 48 hours after plasmid DNA transfection. NCT of S-tdT reporter proteins was quantified as in Fig 2B. Two-way ANOVA detected significant interaction effects of GFP-GR100 and ISRIB treatment on NCT in controlsiRNA cells (P = 0.0097), but not in EPAC1siRNA cells (P = 0.5310 for EPAC1siRNA #1; P = 0.5218 for EPAC1siRNA #2); significant interaction effects of GFP-GR100 and EPAC1 depletion on NCT regardless of ISRIB treatment (P = 0.0194 for EPAC1siRNA #1 in DMSO; P = 0.0133 for EPAC1siRNA #2 in DMSO; P < 0.0001 for both EPAC1siRNA in ISRIB). Data represent means ± SEM (n = 128–140 GFP–or GFP-GR100–positive cells from 3 independent experiments). *P < 0.05, **P < 0.01, ***P < 0.001, as determined by Tukey post hoc test. (E) EPAC1 depletion increases the cell population positive for poly(GR)-induced SGs but suppresses the maturation of poly(GR)-induced SGs. SH-SY5Y cells were co-transfected with siRNA and a GFP-GR100 expression vector, as described in Fig 6C. The assembly of poly(GR)-i [file pbio.3001002.s008.tiff]

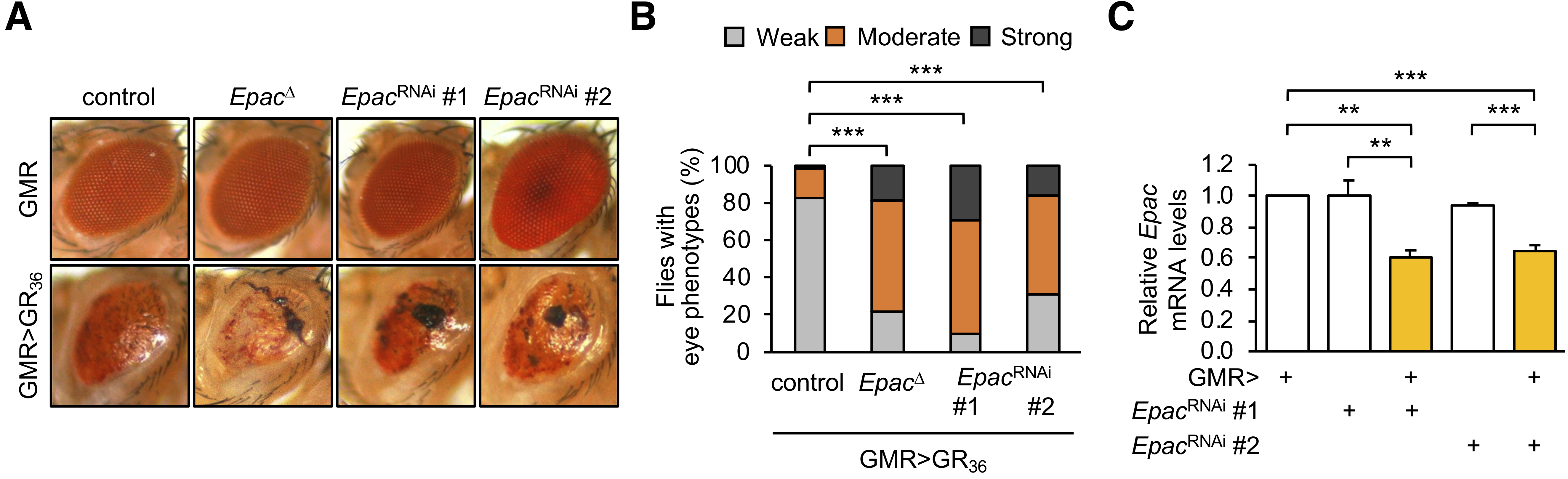

Supplement: S9 Fig — (A) Loss of Epac function exacerbates the neurodegenerative effects of C9ORF72-derived poly(GR) proteins in a Drosophila eye model. Transgenic GR36 protein was overexpressed in photoreceptor neurons (GMR>GR36) of wild-type (control), heterozygous Epac-deletion mutant (EpacΔ), or EPAC-depleted flies (EpacRNAi #1, v50372; EpacRNAi #2, v110077). Representative images of 1-week-old male flies per genotype are shown for ommatidial disorganization and necrotic spots. GMR served as transgenic controls. (B) The eye phenotypes were scored in individual transgenic flies (n = 123–170; weak, moderate, strong), and their relative distribution was calculated for each genotype. ***P < 0.001, as determined by chi-squared test. (C) Two Epac RNAi transgenes (EpacRNAi #1, v50372; EpacRNAi #2, v110077) were individually overexpressed in the Drosophila photoreceptor neurons by GMR-GAL4 driver. Total RNA was prepared from fly heads. The abundance of Epac transcripts was quantified by real-time RT-PCR and normalized to that of poly(A)-binding protein. Relative Epac mRNA levels were then calculated by normalizing to those in GMR-GAL4/+ heterozygous controls. Data represent means ± SEM (n = 3). *P < 0.05, ***P < 0.001, as determined by 1-way ANOVA with Dunnett post hoc test. All underlying numerical values are available in S1 Data. ANOVA, analysis of variance; EPAC, exchange protein directly activated by cyclic AMP 1; GMR, glass multiple reporter; RT-PCR, reverse transcription PCR; SEM, standard error of the mean. (TIFF) [file pbio.3001002.s009.tiff]

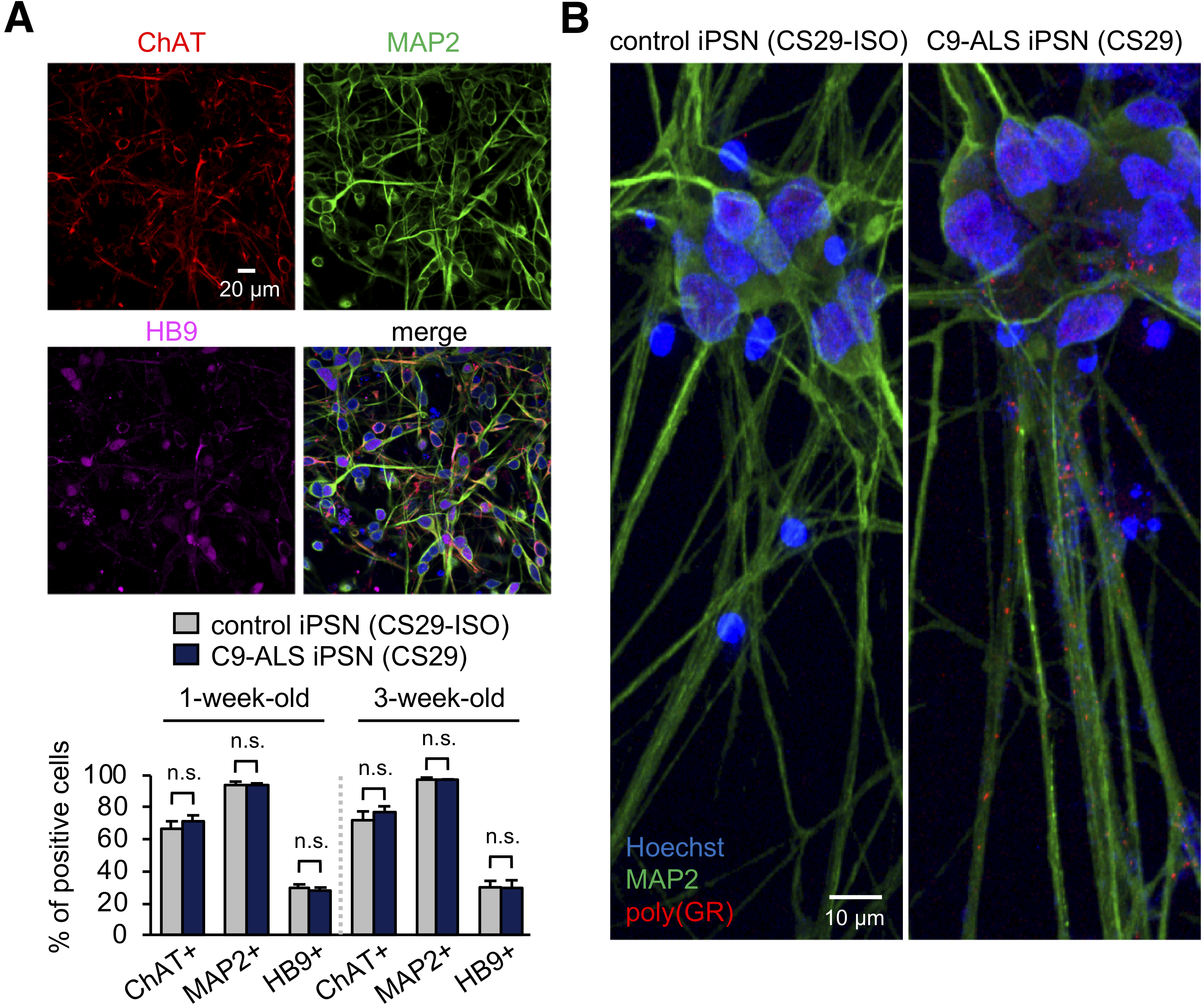

Supplement: S10 Fig — (A) C9-ALS iPSNs (CS29) and isogenic control iPSNs (CS29-ISO) were fixed 7 days (1-week-old) or 21 days (3-week-old) after neuronal differentiation from their parental NPCs. Immunofluorescence assays were performed using anti-ChAT antibody (red), anti-MAP2 antibody (green), anti-HB9 antibody (magenta), and Hoechst 33258 (blue). The percentages of MAP2-positive (a neuronal marker), ChAT-positive (cholinergic neurons), and HB9-positive cells (motor neurons) were calculated and averaged (n = 7 confocal images from 3 independent experiments; n = 511–624 Hoechst–positive cells). Error bars indicate SEM. n.s., not significant; as determined by 2-way ANOVA with Tukey post hoc test. (B) poly(GR) aggregates are readily detectable in C9-ALS iPSNs, but not isogenic control neurons. iPSNs were co-stained with anti-MAP2 antibody (green), anti-poly(GR) antibody (red), and Hoechst 33258 (blue) similarly as above. All underlying numerical values are available in S1 Data. ANOVA, analysis of variance; C9-ALS, C9ORF72-associated amyotrophic lateral sclerosis; NPC, neural progenitor cell; SEM, standard error of the mean. (TIFF) [file pbio.3001002.s010.tiff]

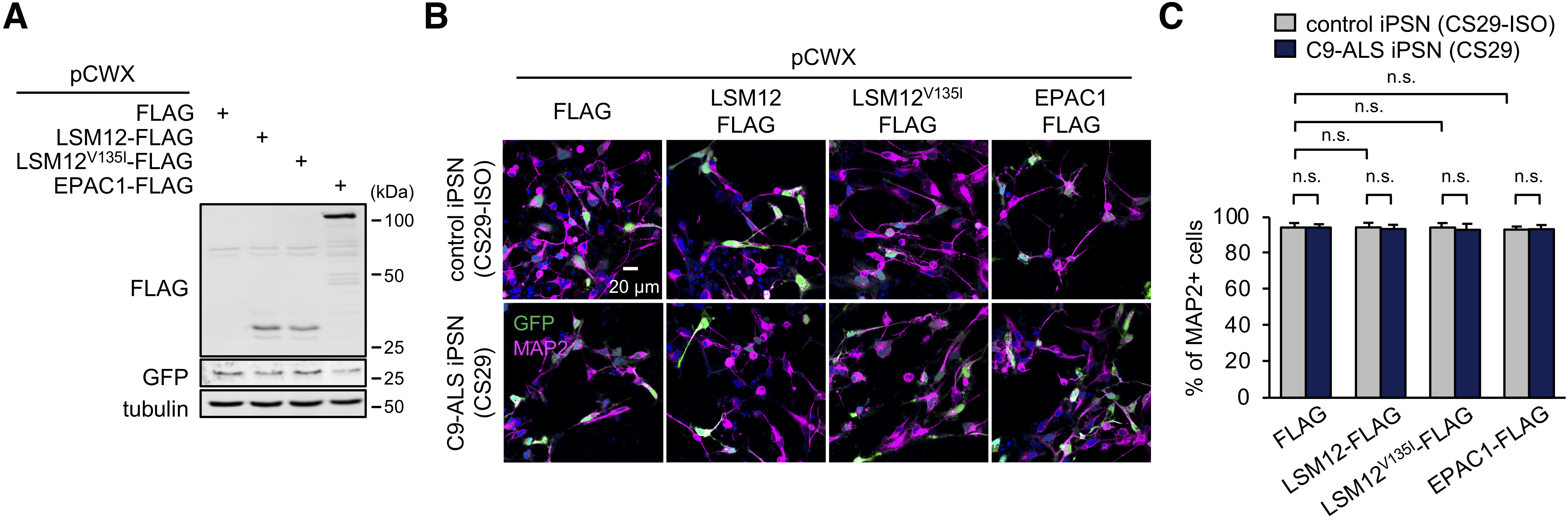

Supplement: S11 Fig — (A) SH-SY5Y cells were infected with individual recombinant lentiviruses that express the indicated FLAG-tagged proteins along with a GFP reporter. Total cell extracts were prepared 48 hours after infection and immunoblotted with anti-FLAG, anti-GFP, and anti-tubulin (loading control) antibodies. (B) NPCs from C9-ALS iPSCs (CS29) and their isogenic control cells (CS29-ISO) were transduced with the indicated recombinant lentiviruses. Three-week-old iPSNs were fixed and co-stained with anti-MAP2 antibody (magenta) and Hoechst 33258 (blue). (C) The percentages of MAP2–positive neurons among GFP–positive cells were calculated and averaged (n = 8 confocal images obtained from 4 independent experiments; n = 123–156 GFP–positive cells). Error bars indicate SEM. n.s., not significant; as determined by 2-way ANOVA with Tukey post hoc test. All underlying numerical values are available in S1 Data. ANOVA, analysis of variance; EPAC1, exchange protein directly activated by cyclic AMP 1; GFP, green fluorescent protein; iPSC, induced pluripotent stem cell; LSM12, like-Sm protein 12; NPC, neural progenitor cells; SEM, standard error of the mean. (TIFF) [file pbio.3001002.s011.tiff]

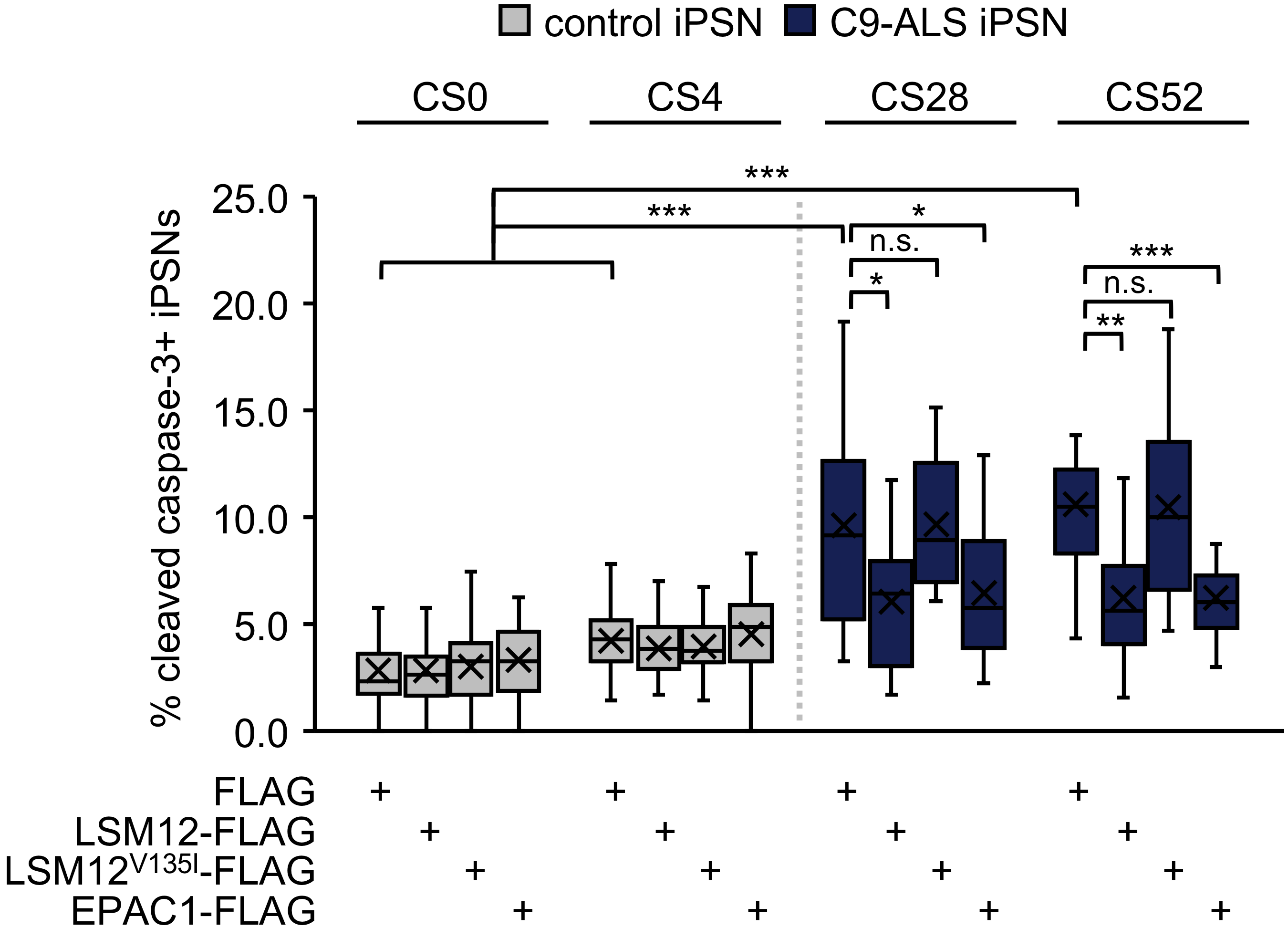

Supplement: S12 Fig — Three-week-old C9-ALS iPSNs (CS28 and CS52) and control iPSNs (CS0 and CS4) were fixed and co-stained with anti-cleaved caspase-3 antibody, anti-MAP2 antibody, and Hoechst 33258. The relative percentages of iPSNs expressing cleaved caspase-3 were quantified as in Fig 7H. Data represent means ± SEM (n = 828–1,122 GFP-positive cells from 3 independent differentiation experiments). n.s., not significant; *P < 0.05, **P < 0.01, ***P < 0.001, as determined by 2-way ANOVA with Tukey post hoc test. All underlying numerical values are available in S1 Data. ANOVA, analysis of variance; C9-ALS, C9ORF72-associated amyotrophic lateral sclerosis; EPAC1, exchange protein directly activated by cyclic AMP 1; GFP, green fluorescent protein; LSM12, like-Sm protein 12; SEM, standard error of the mean. (TIFF) [file pbio.3001002.s012.tiff]

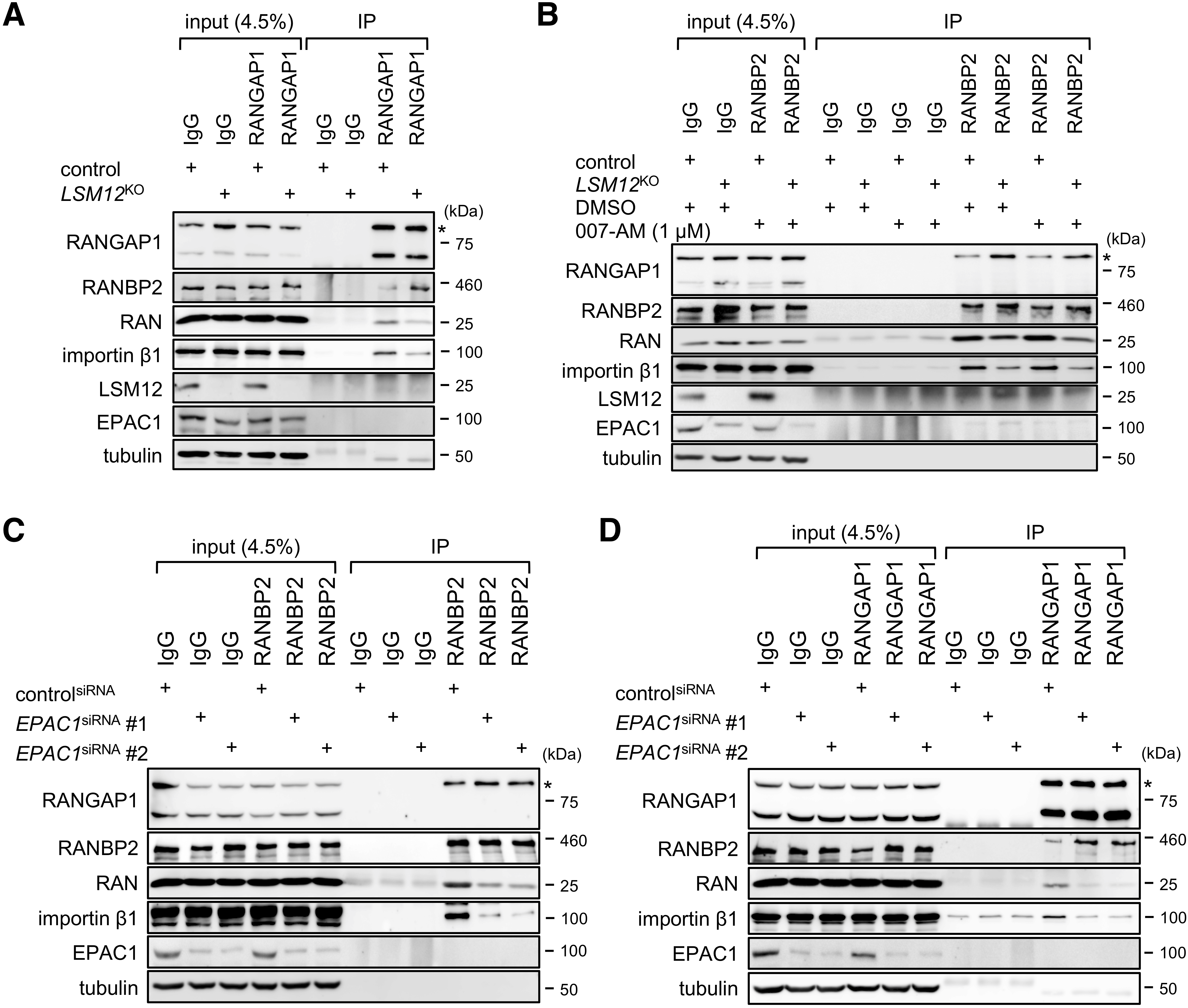

Supplement: S13 Fig — (A) LSM12 deletion dissociates RAN and importin β1 from the RANBP2-RANGAP1 complex. Soluble extracts from control or LSM12KO cells were immunoprecipitated with control IgG or anti-RANGAP1 antibody. IP complexes were analyzed as in Fig 8A. Asterisks indicate SUMOylated RANGAP1. Input, 4.5% of soluble extracts used in each IP. (B) A selective activator of EPAC1 (8-pCPT-2′-O-Me-cAMP-AM/007-AM) does not rescue the association of RAN and importin β1 with the RANBP2-RANGAP1 complex in LSM12-deleted cells. Where indicated, cells were preincubated with 1-μM 007-AM or DMSO (vehicle control) at 37°C for 1 hour before immunoprecipitating soluble cell extracts with control IgG or anti-RANBP2 antibody. (C, D) EPAC1 depletion dissociates RAN and importin β1 from the RANBP2-RANGAP1 complex. Soluble extracts from control or EPAC1 siRNA-transfected cells were immunoprecipitated with anti-RANBP2 (C) or anti-RANGAP1 antibodies (D). EPAC1, exchange protein directly activated by cyclic AMP 1; IP, immunoprecipitation; LSM12, like-Sm protein 12; RAN, repeat-associated non-AUG; RANBP2, Ran-binding protein 2; RANGAP1, RAN GTPase-activating protein 1. (TIFF) [file pbio.3001002.s013.tiff]

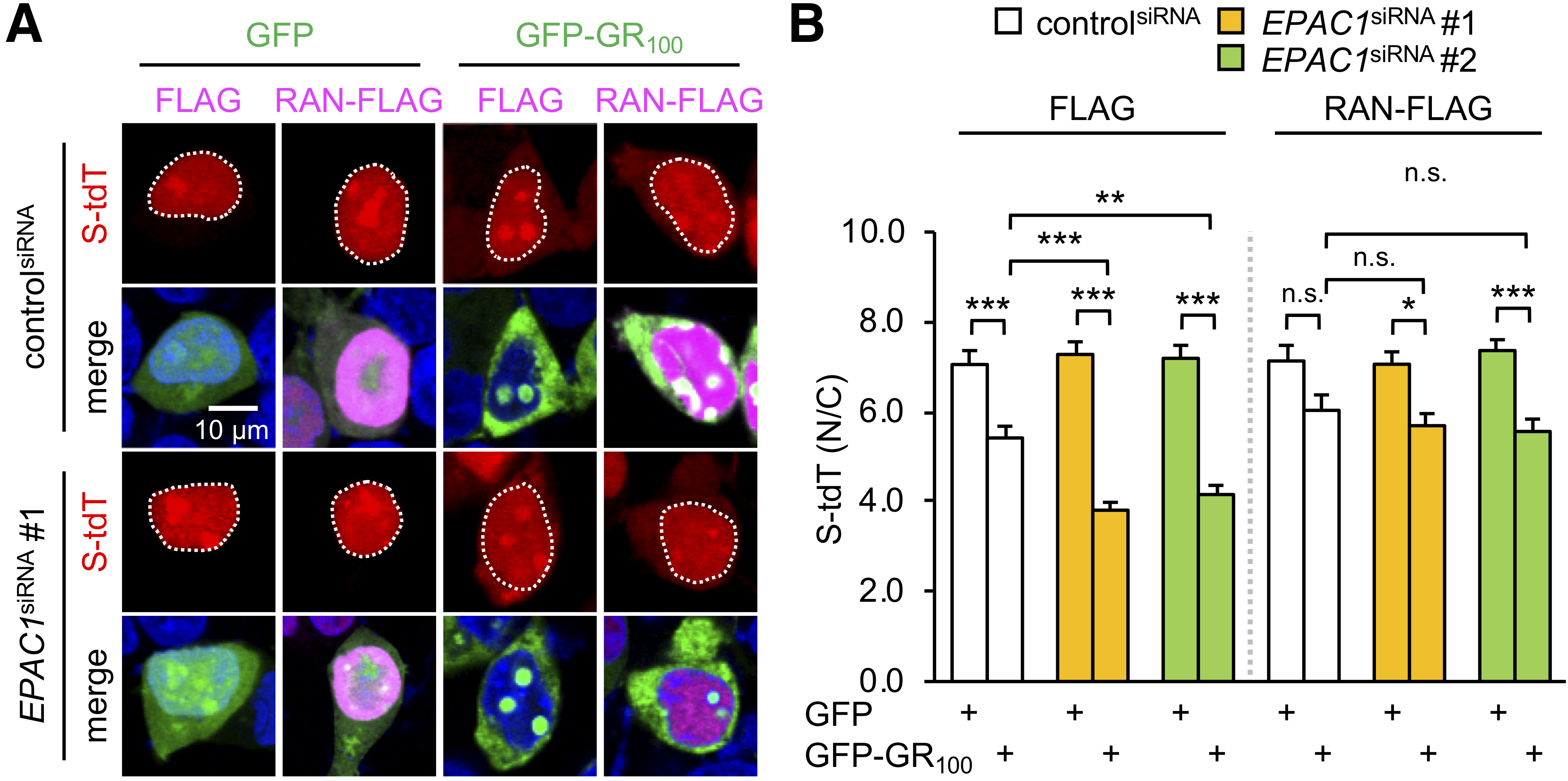

Supplement: S14 Fig — (A) SH-SY5Y cells were co-transfected with siRNA and expression vectors for S-tdT, GFP-GR100, and RAN-FLAG, as in Fig 6A. Transfected cells were co-stained with anti-FLAG antibody (magenta) and Hoechst 33258 (blue) 48 hours after plasmid DNA transfection. (B) NCT of S-tdT reporter proteins was quantified as in Fig 2A. Two-way ANOVA detected significant interaction effects of GFP-GR100 and EPAC1 depletion on NCT only in FLAG-expressing cells (P = 0.0005 for EPAC1siRNA #1; P = 0.0093 for EPAC1siRNA #2). Data represent means ± SEM (n = 113–115 GFP–or GFP-GR100–positive cells from 3 independent experiments). n.s., not significant; *P < 0.05, **P < 0.01, ***P < 0.001, as determined by Tukey post hoc test. All underlying numerical values are available in S1 Data. GFP, green fluorescent protein; NCT, nucleocytoplasmic transport; RAN, repeat-associated non-AUG; S-tdT, S-tdTomato; SEM, standard error of the mean; siRNA, small interfering RNA. (TIFF) [file pbio.3001002.s014.tiff]

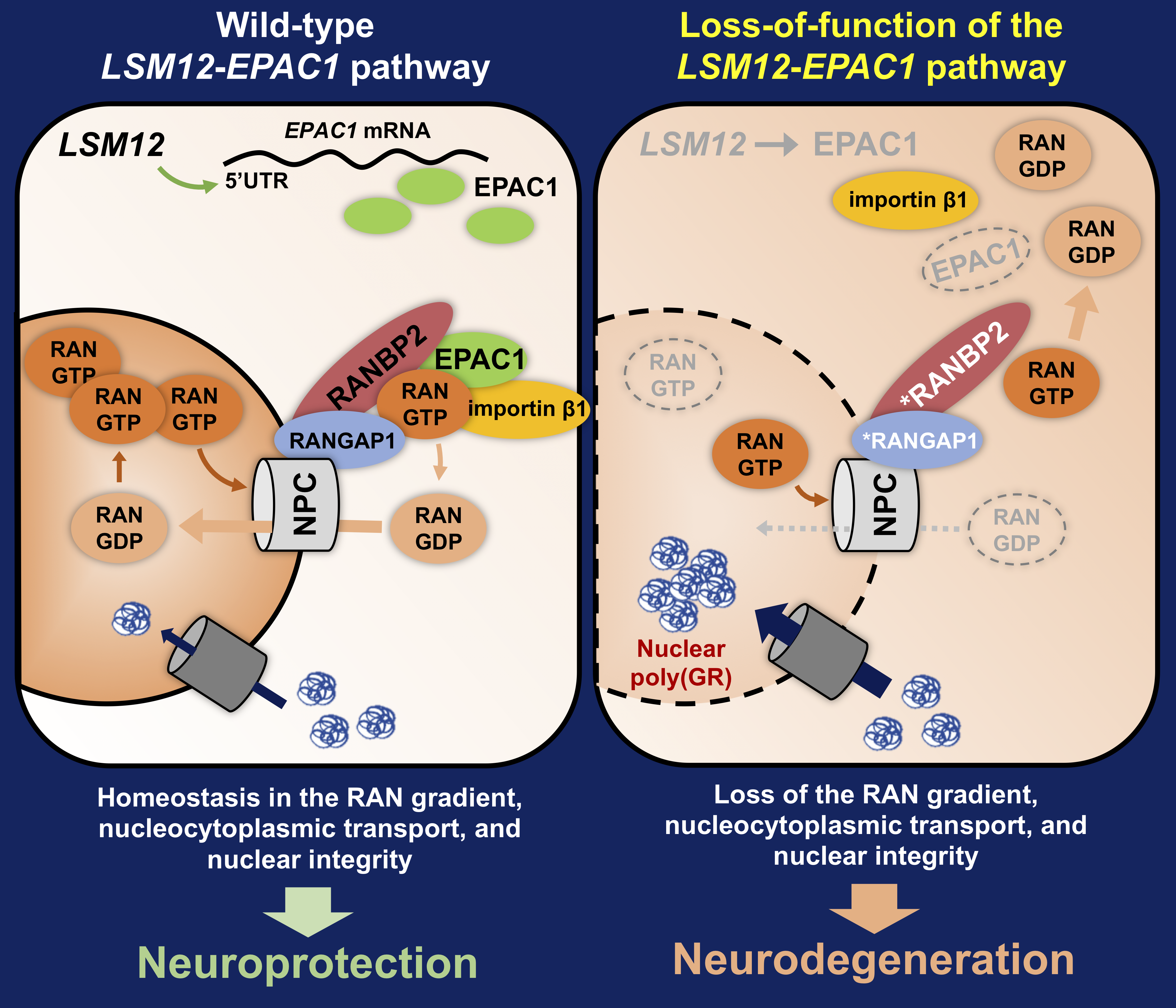

Supplement: S15 Fig — The LSM12-EPAC1 pathway contributes to the assembly of the RAN-associating protein complex at the cytoplasmic side of the nuclear pore, thereby facilitating RAN-GTP recycling. The loss-of-function of the LSM12-EPAC1 pathway dissociates RAN and importin β1 from the RANBP2-RANGAP1 complex and delays the nuclear entry of RAN for recycling. Disruption of the RAN gradient impairs NCT, promotes the nuclear assembly of poly(GR) granules, and increases poly(GR) toxicity (e.g., loss of nuclear laminar integrity and degeneration of Drosophila photoreceptor neurons). Asterisks indicate stronger interaction between RANBP2 and RANGAP1 caused by the loss of the LSM12-EPAC1 pathway. All underlying numerical values are available in S1 Data. EPAC1, exchange protein directly activated by cyclic AMP 1; LSM12, like-Sm protein 12; RAN, repeat-associated non-AUG; RANBP2, Ran-binding protein 2; RANGAP1, RAN GTPase-activating protein 1. (TIFF) [file pbio.3001002.s015.tiff]

**Fig 1C**

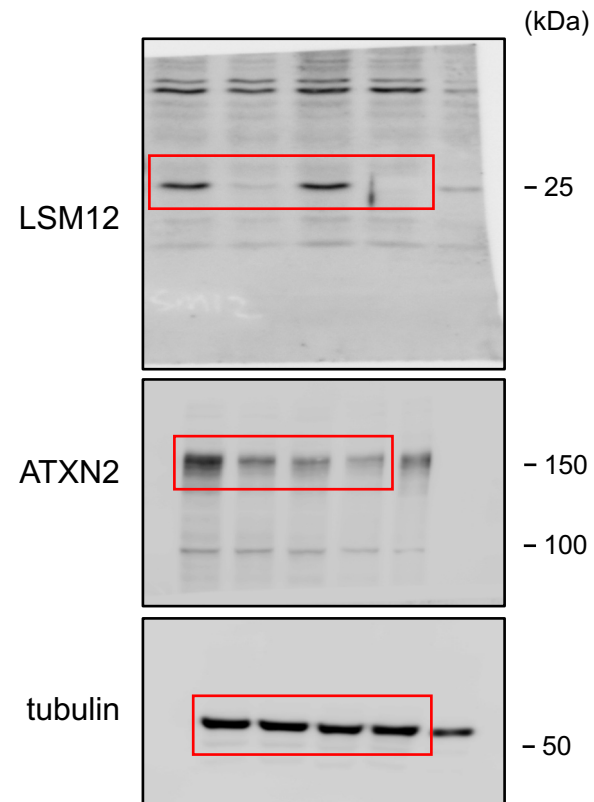

**Fig 5C**

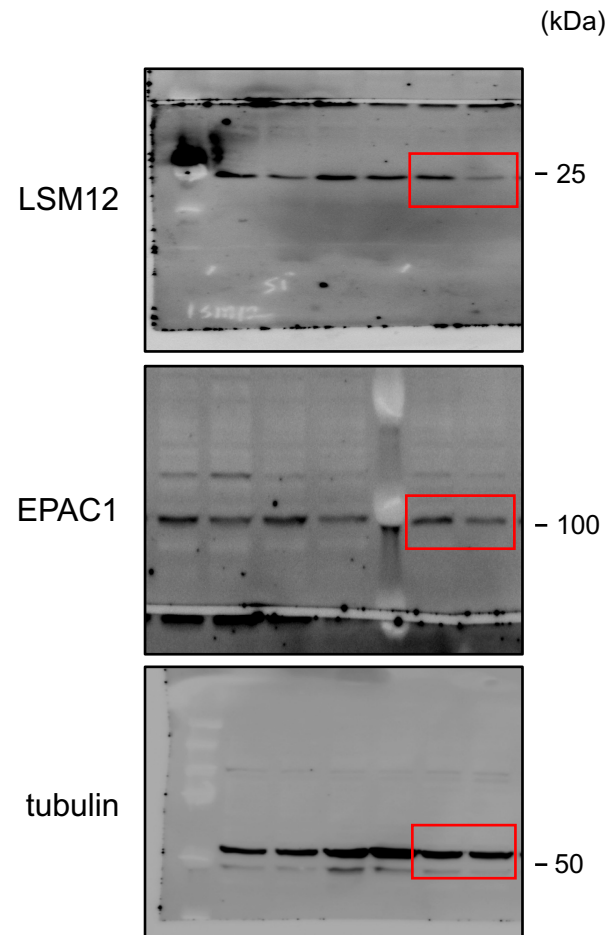

**Fig 7A**

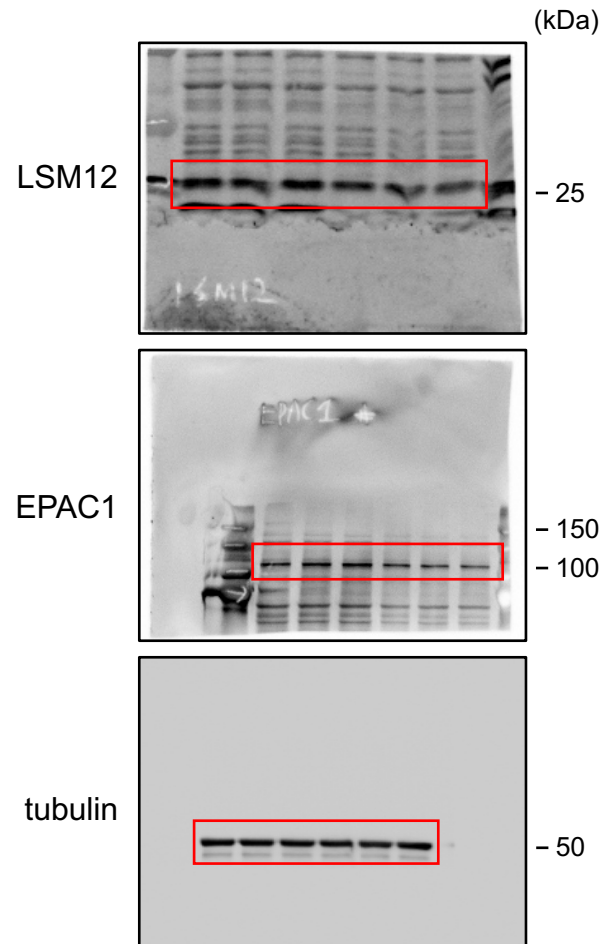

**Fig 8A**

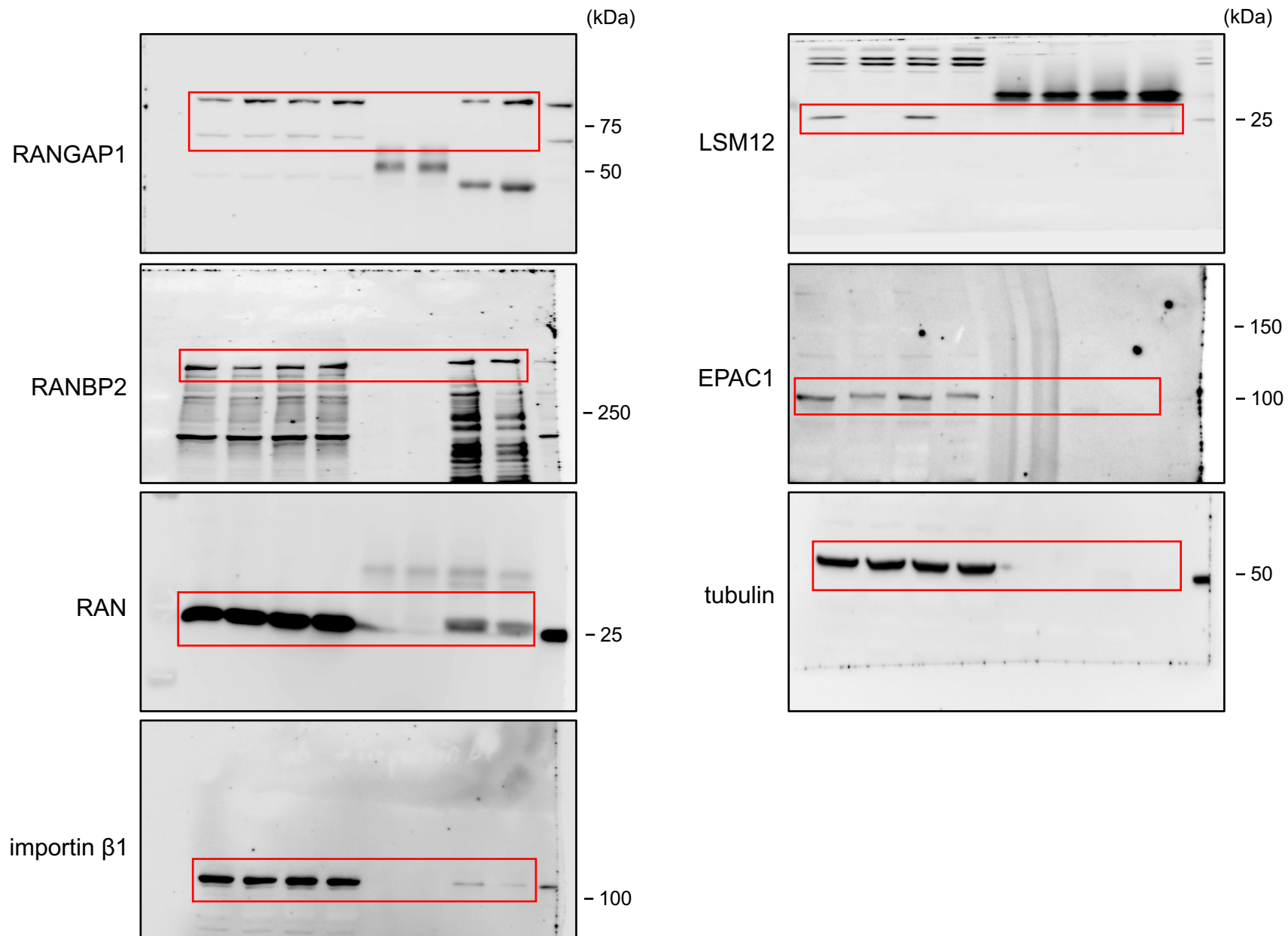

**Fig 8B**

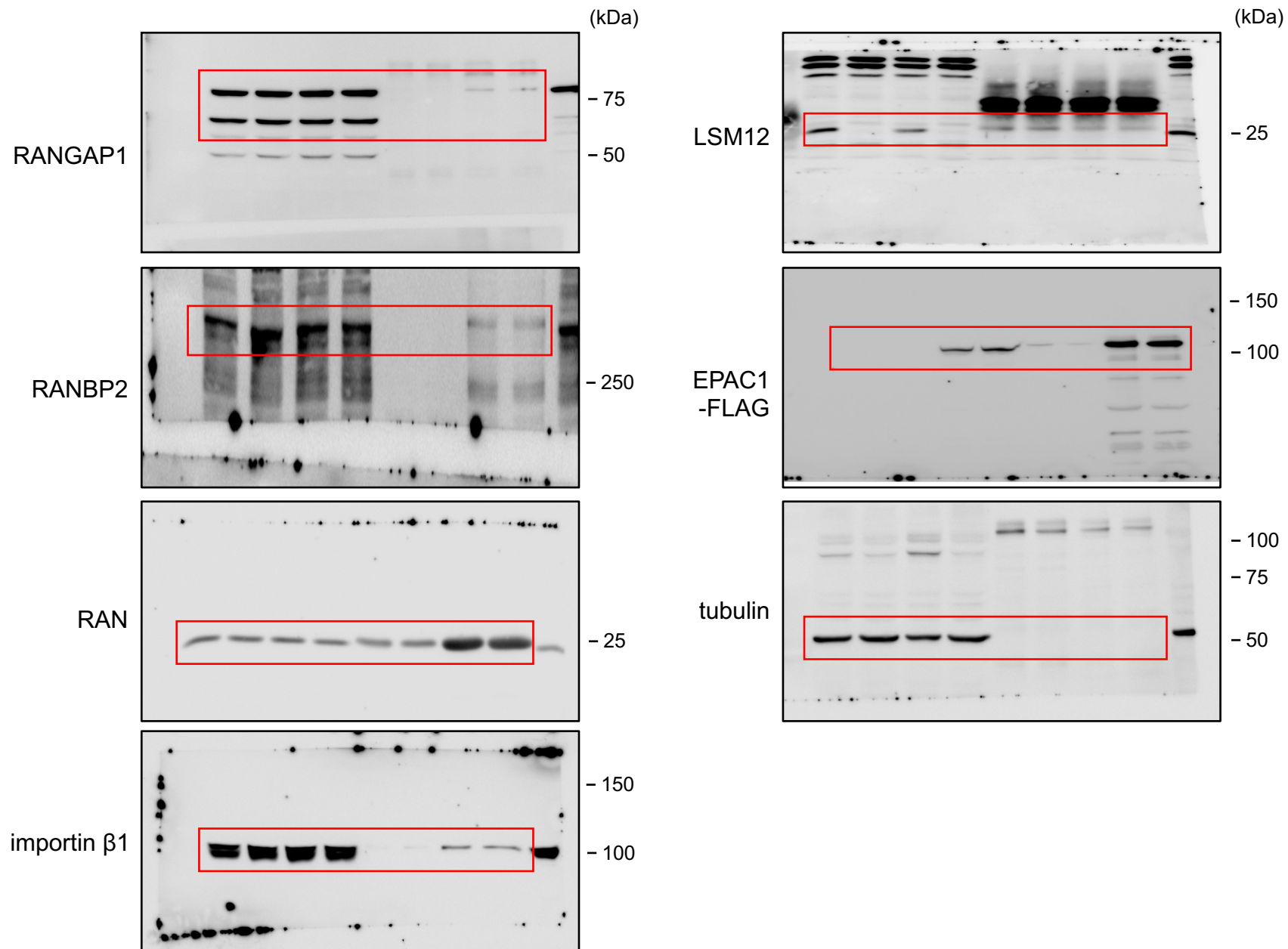

**Fig 8C**

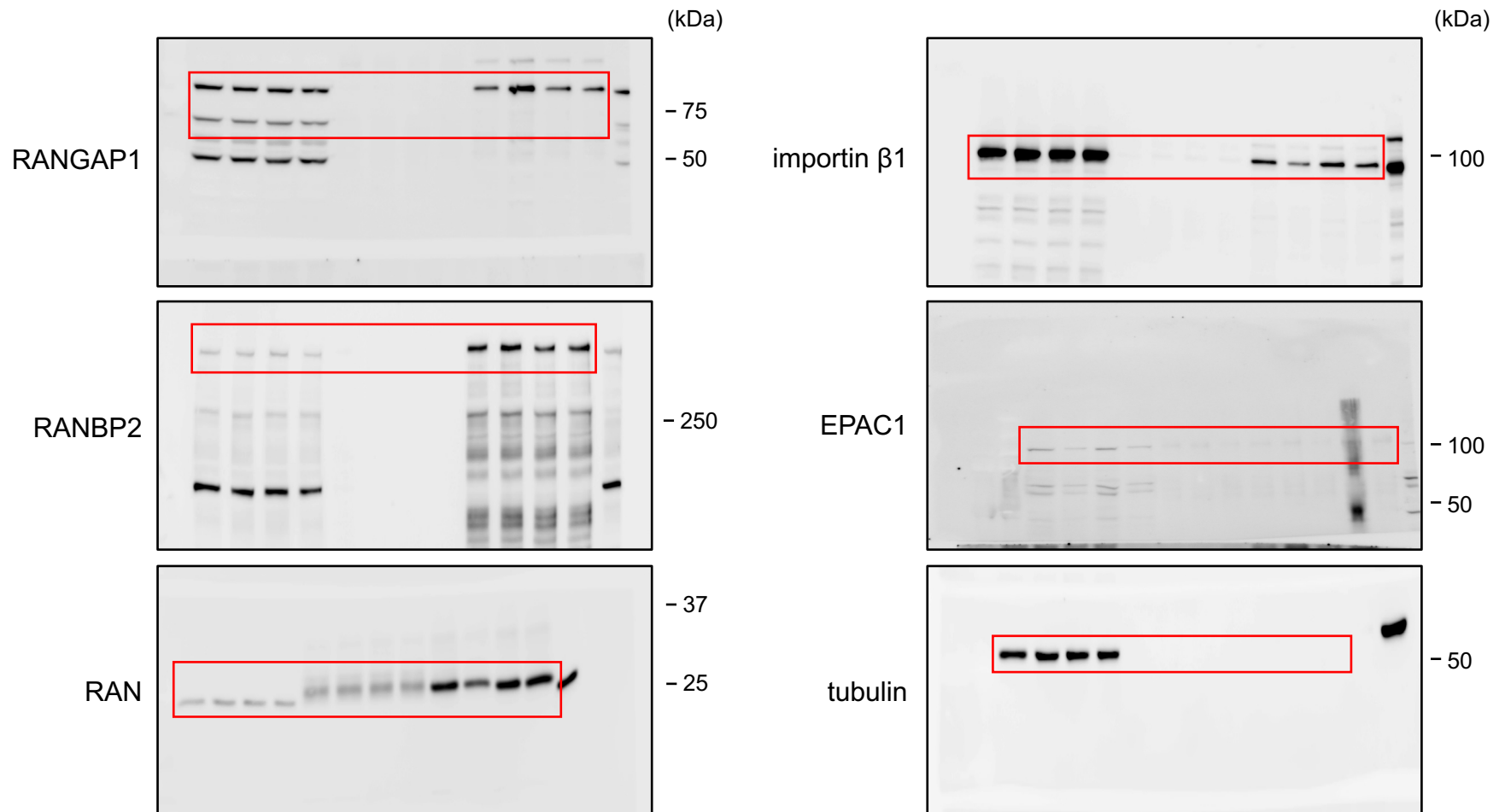

S2B Fig

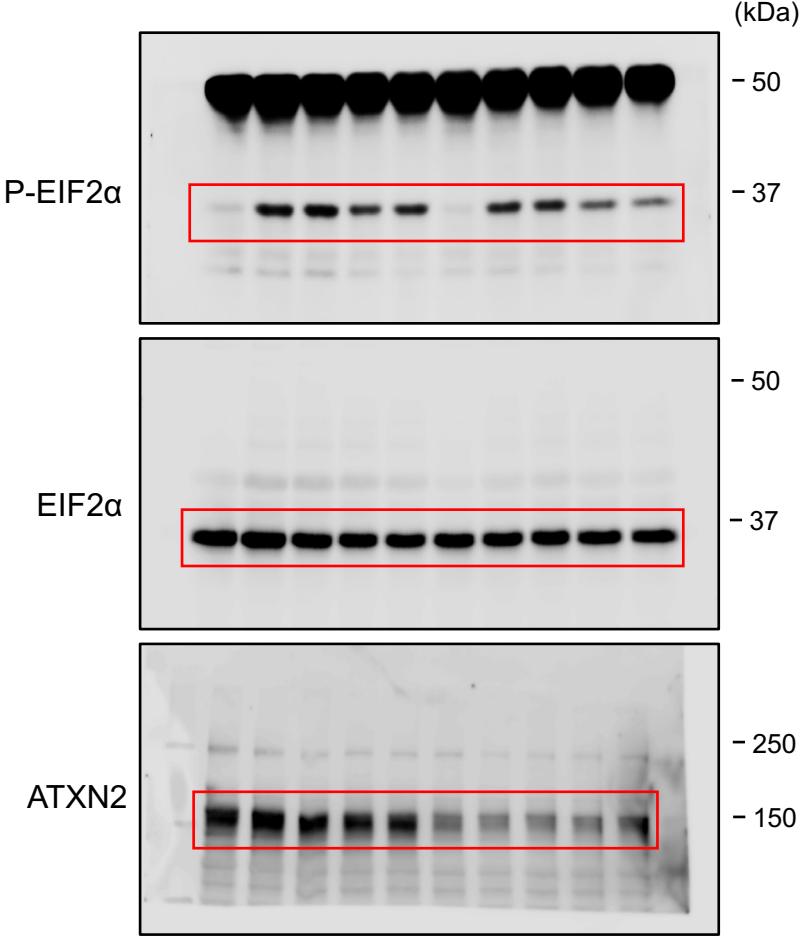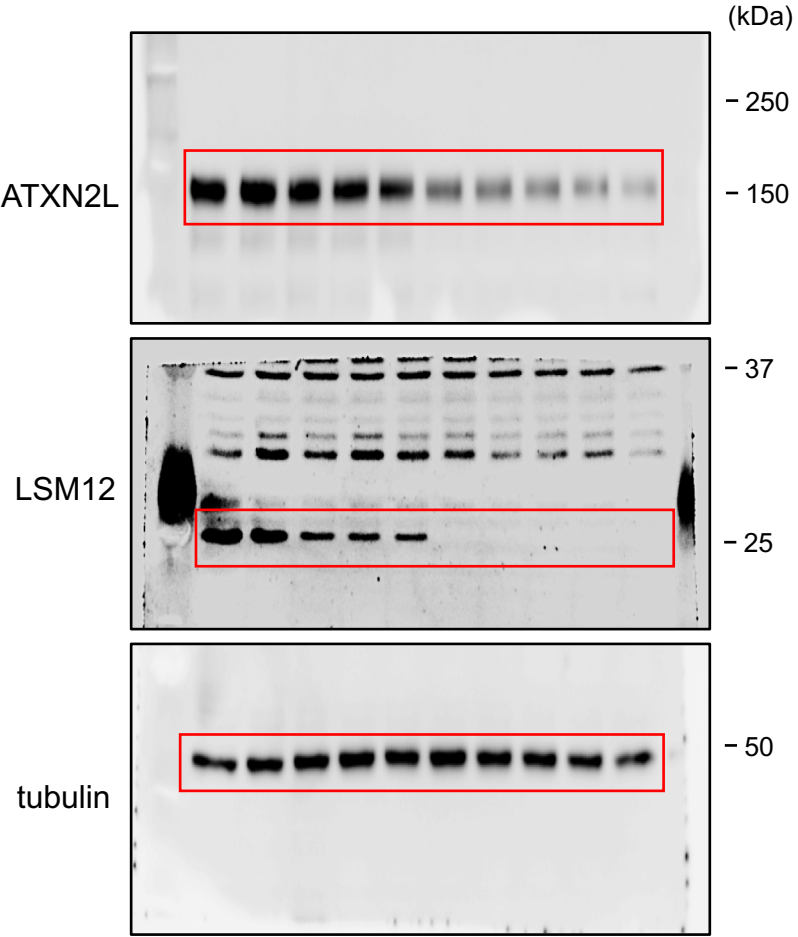

S2C Fig

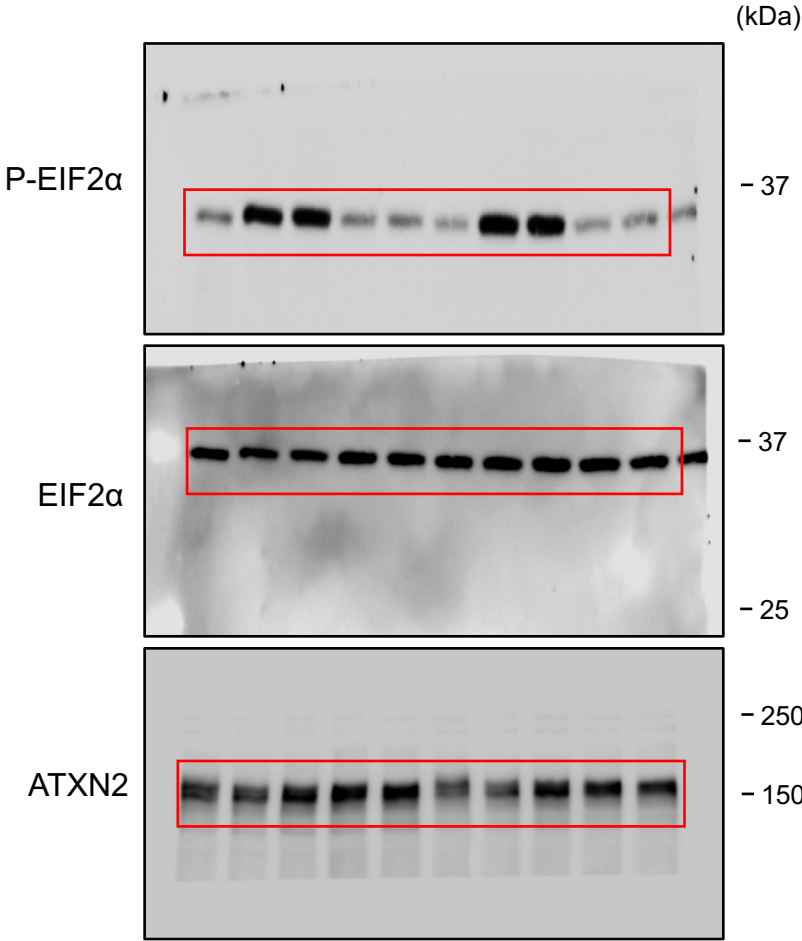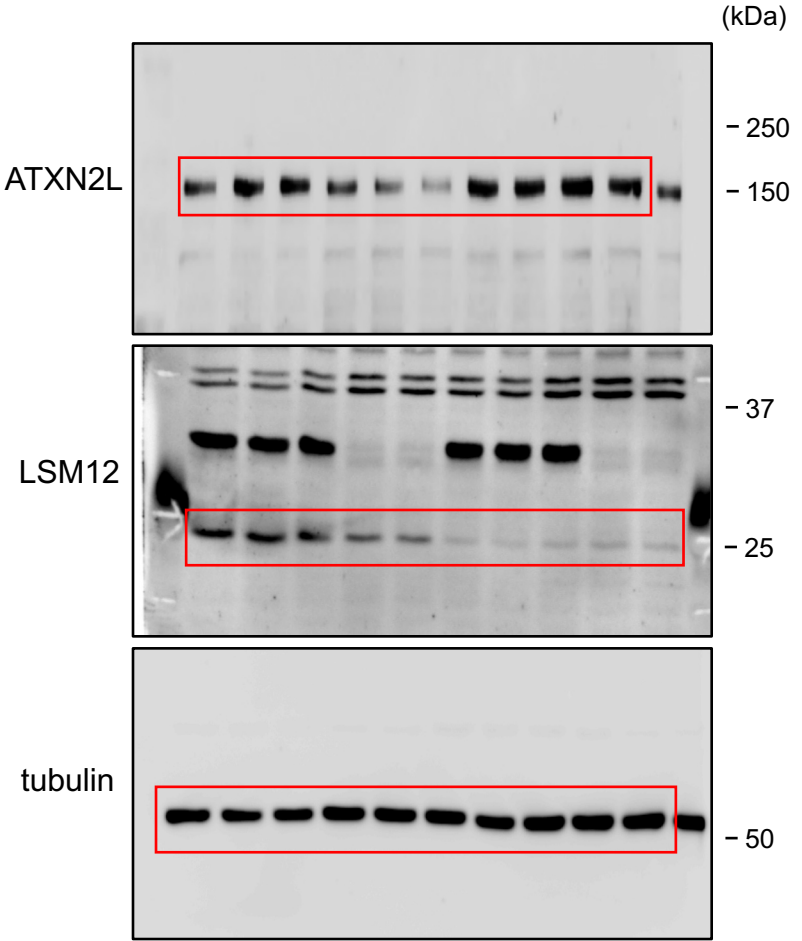

**S5A Fig**

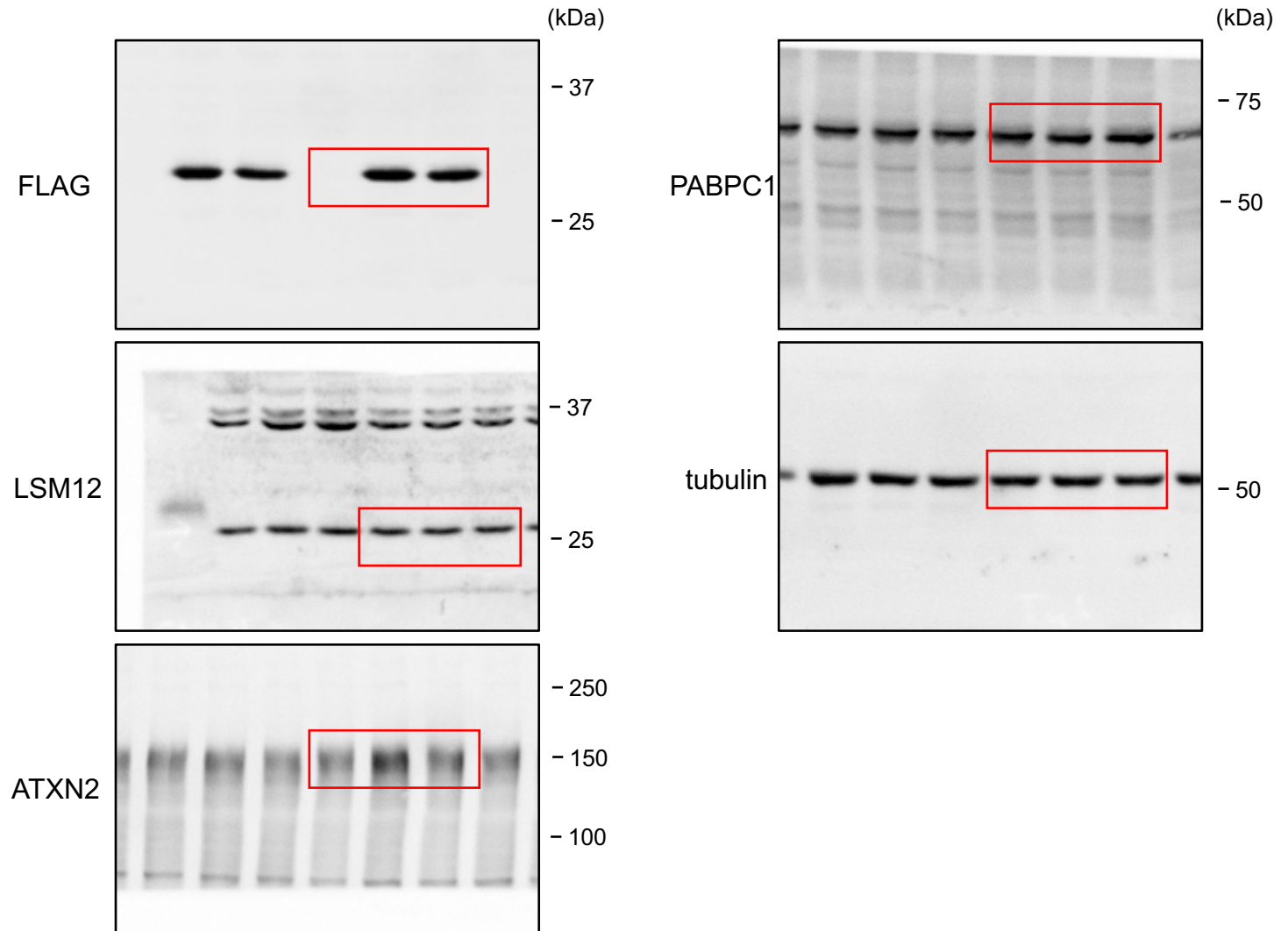

**S7A Fig**

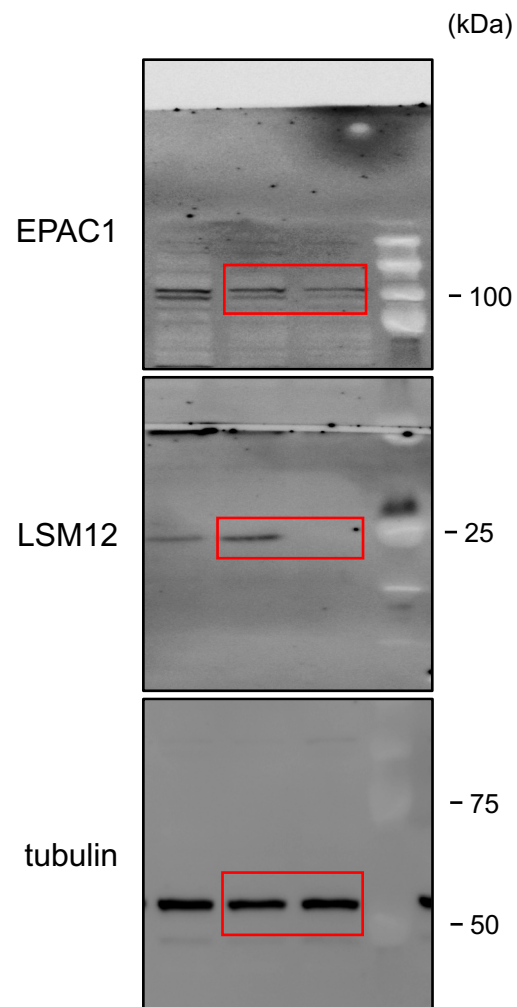

**S8A Fig**

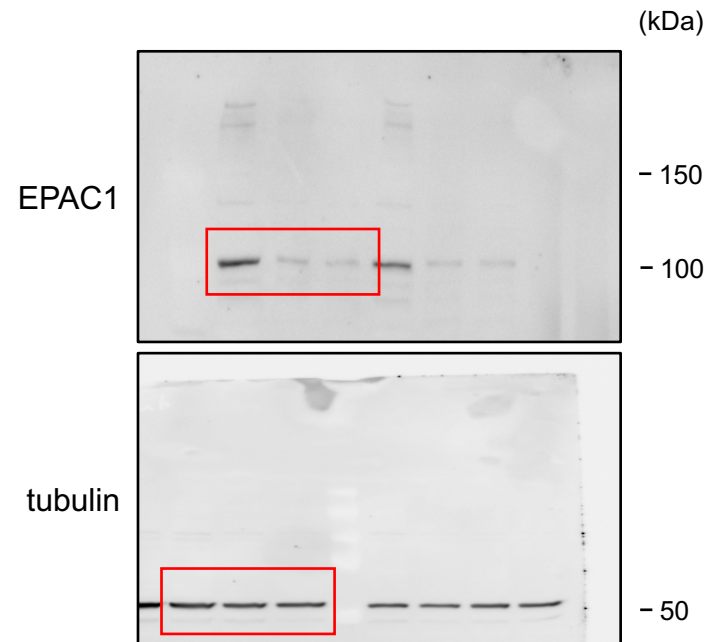

# S11A Fig

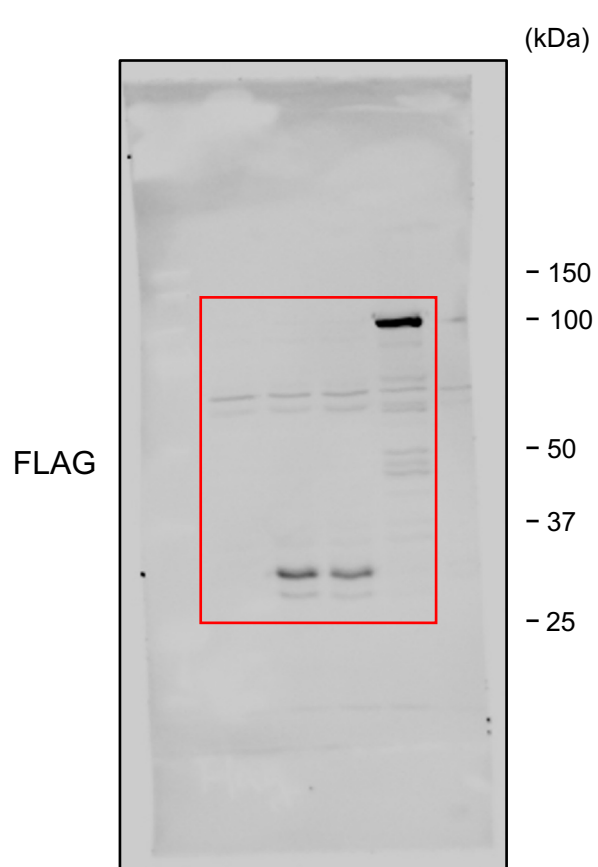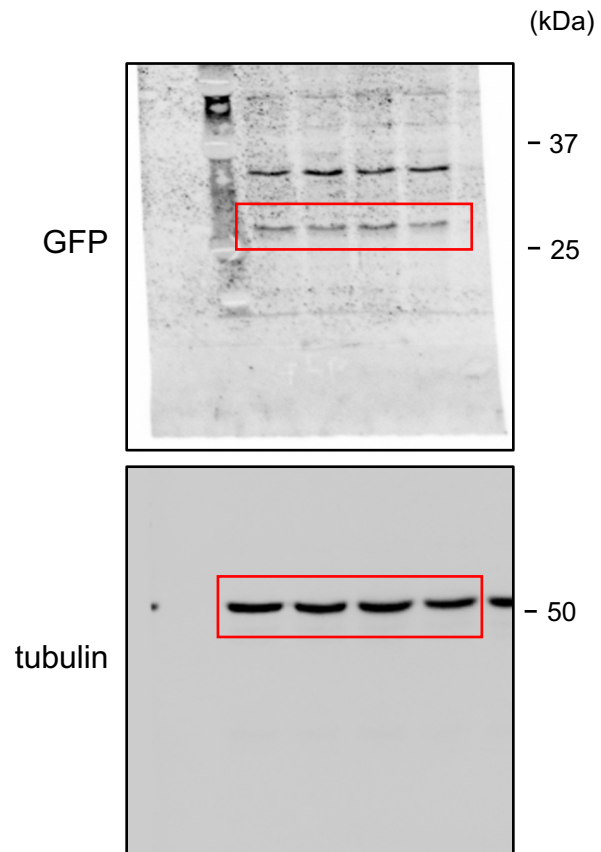

# S13A Fig

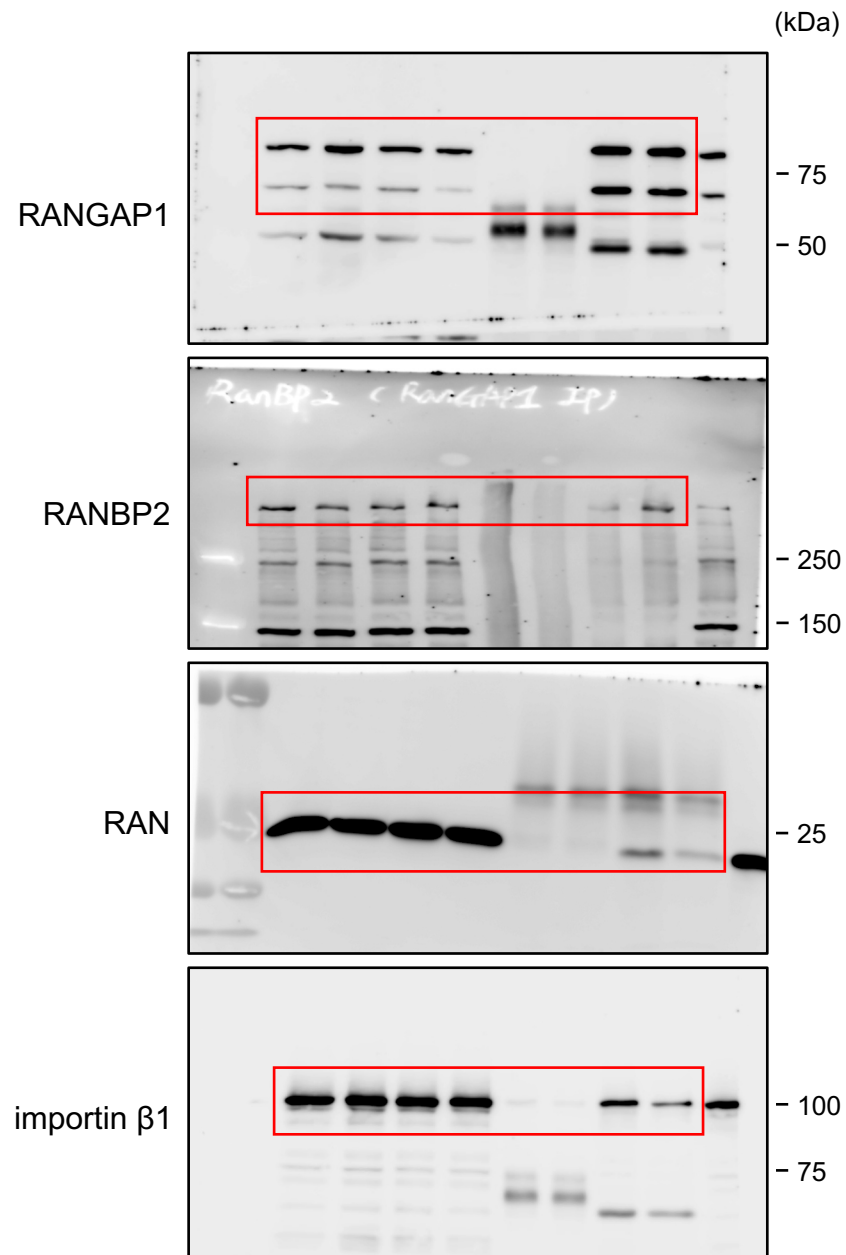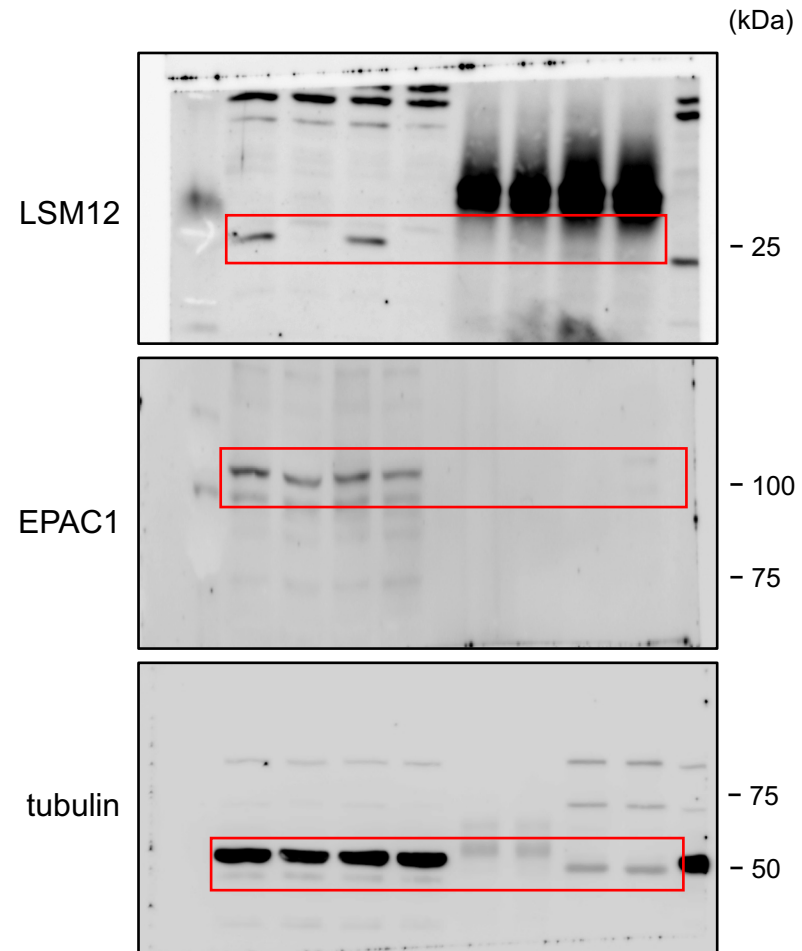

# S13B Fig

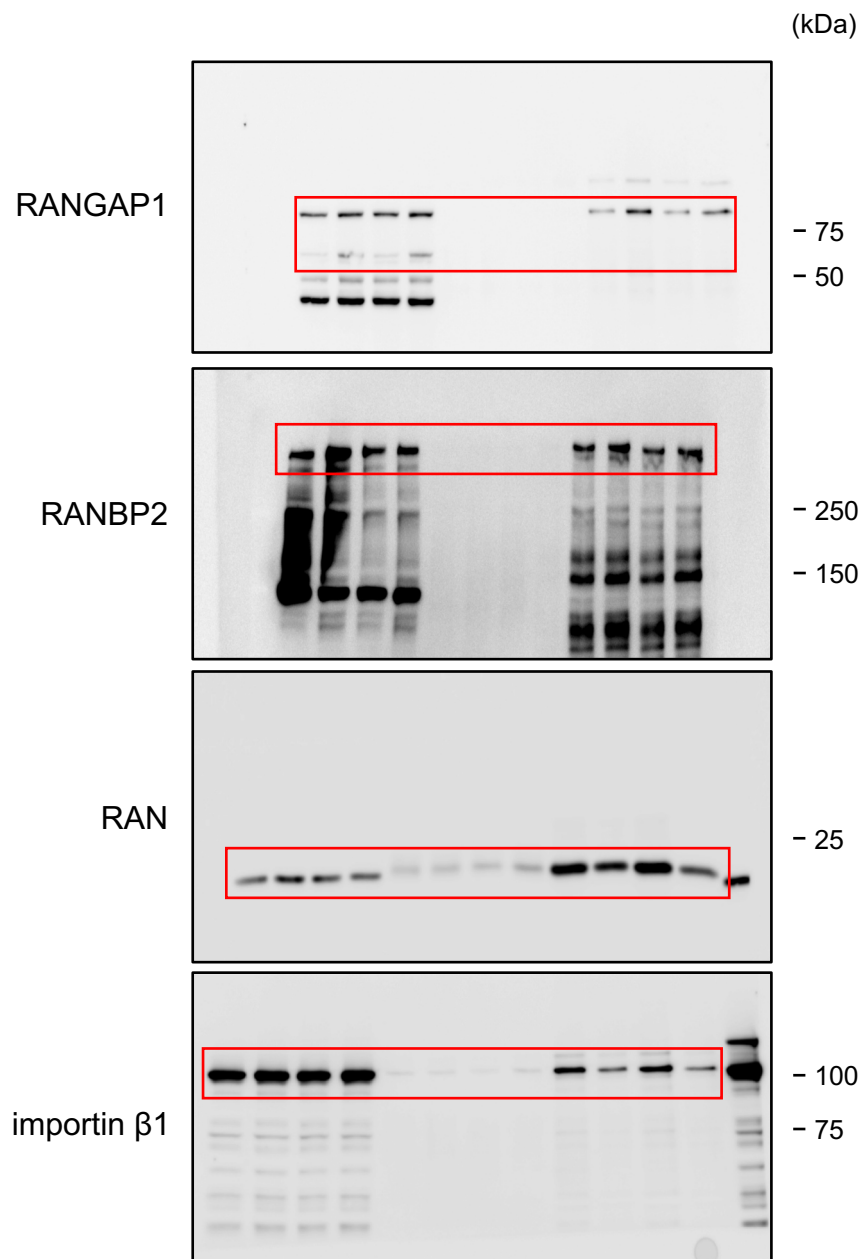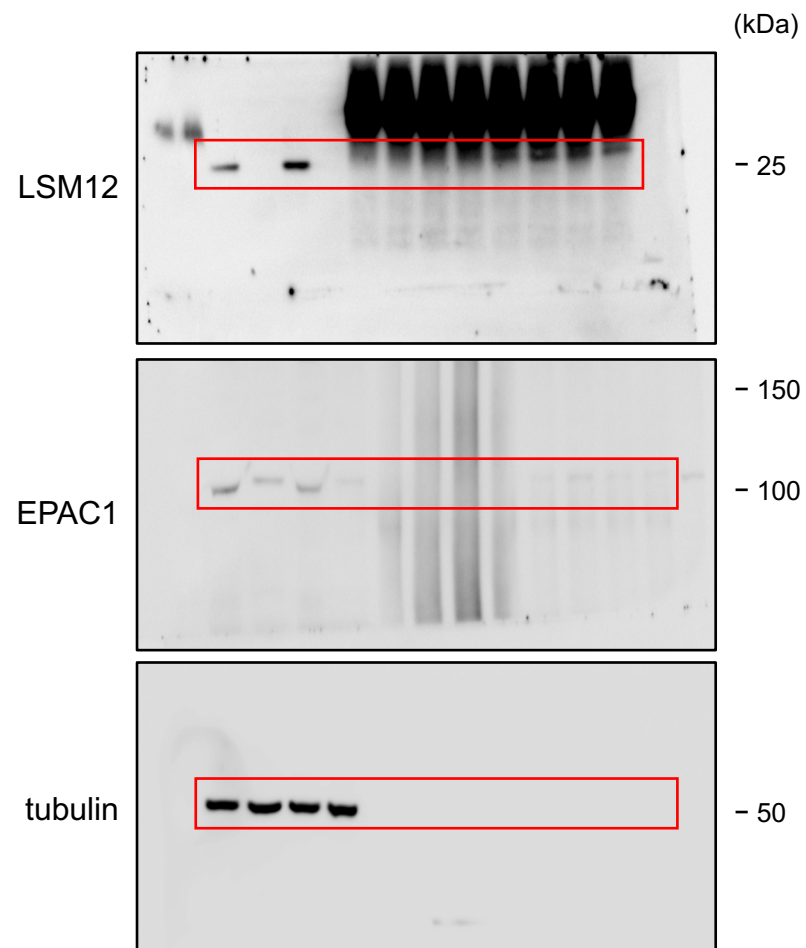

# S13C Fig

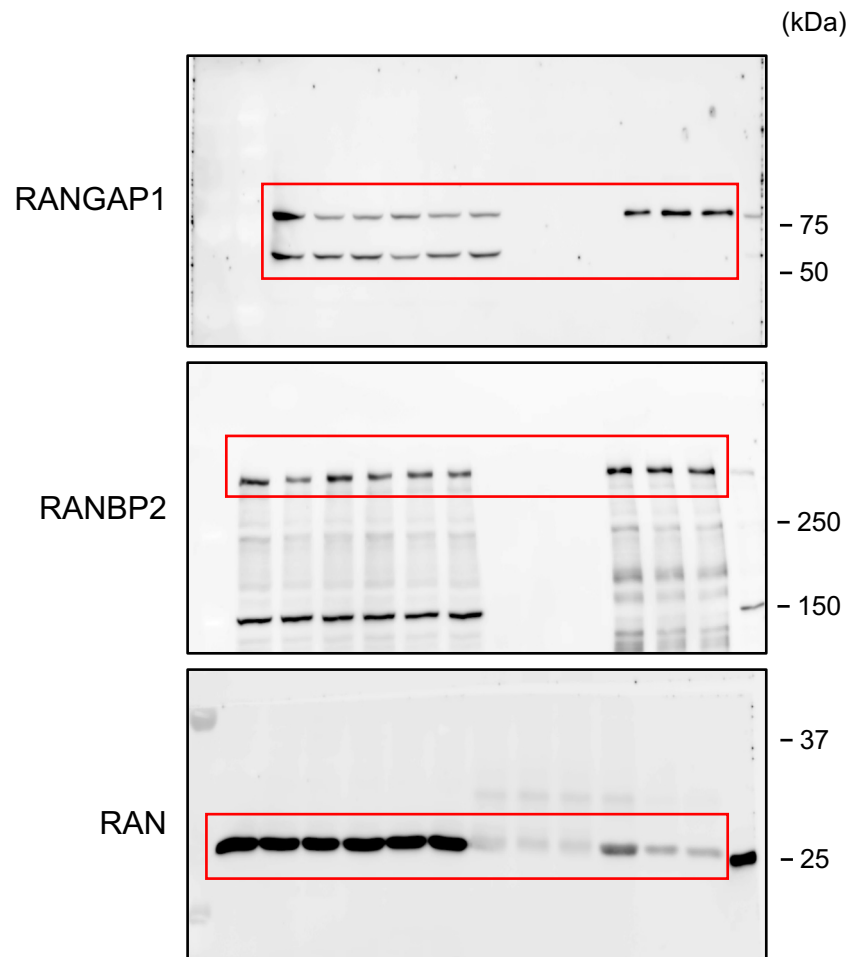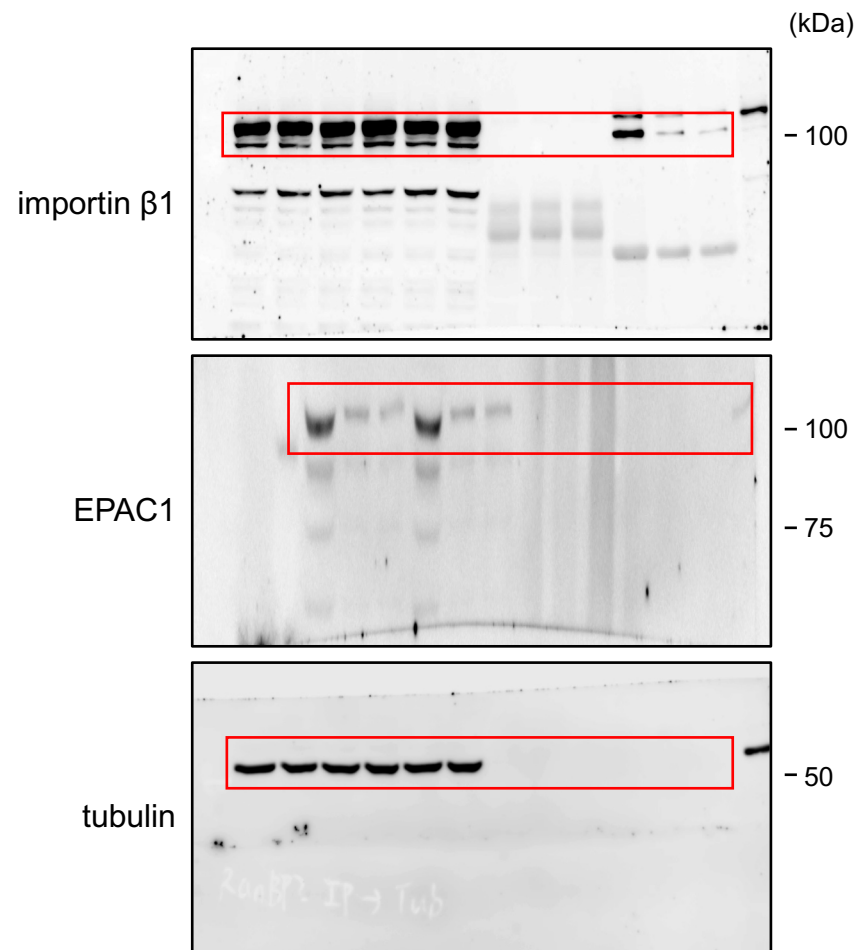

# S13D Fig

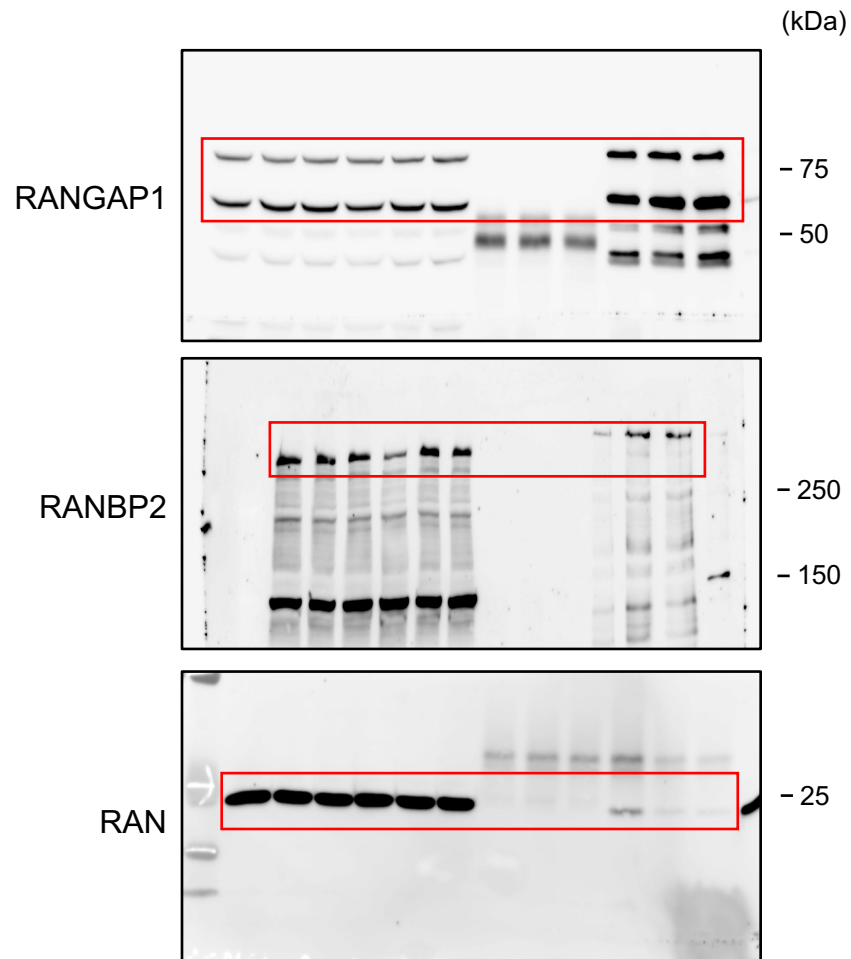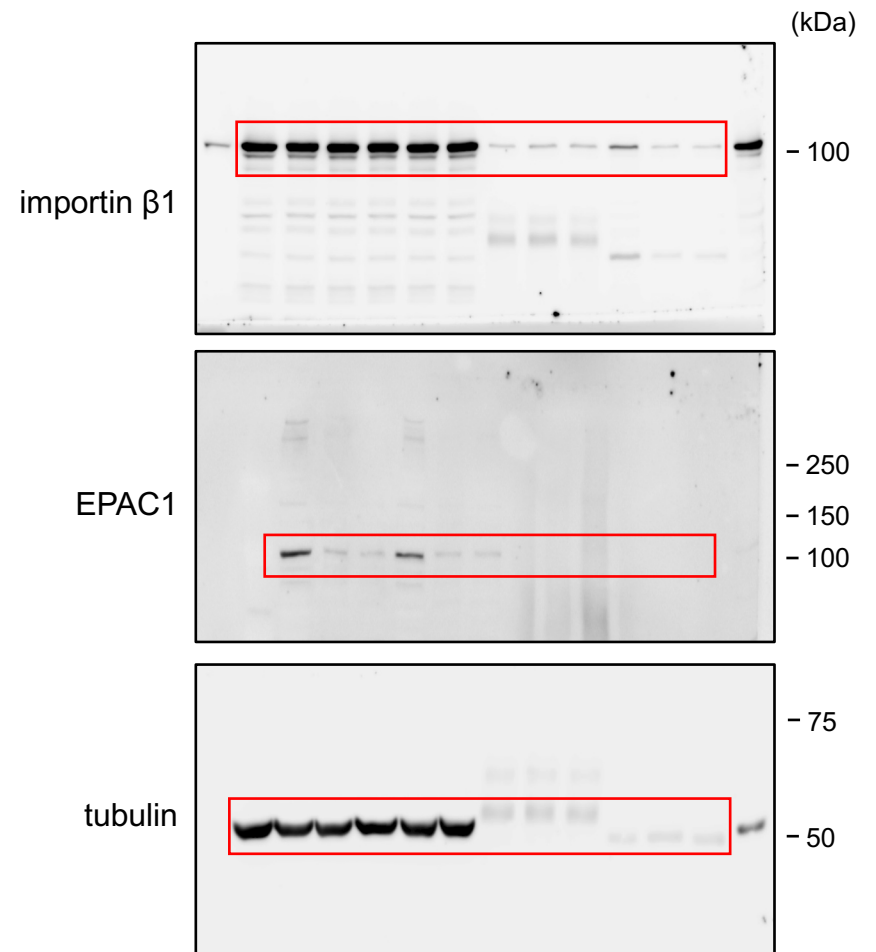

Supplement: S1 Raw Images — (PDF) [file pbio.3001002.s017.pdf]
