# Supplementary material for: LSM12-EPAC1 defines a neuroprotective pathway that sustains the nucleocytoplasmic RAN gradient
Source: PLoS Biol. 2020 Dec 23;18(12):e3001002. doi: 10.1371/journal.pbio.3001002 (PMC7757817; doi:10.1371/journal.pbio.3001002)
Supplement: Attachment — Submitted filename: Response To Reviewers.pdf [file pbio.3001002.s020.pdf]

Please find our point-by-point responses below in blue.

Reviewers' comments

Rev. 1:

The authors have addressed all my concerns. I am happy with the manuscript in its form.

Rev. 2:

This manuscript from Lee and colleagues is much improved from the initial submission. Unfortunately, though, the new and potentially exciting data in iPS neurons does not meet minimum standards of rigor for publication. Only a single C9 iPS cell line is used, far less than the typical 5 line minimum considered standard in the field. Furthermore, there is no data in this cell line characterizing the number of G4C2 repeats in the C9-HRE line or in the corrected c9orf72 gene (how many G4C2 repeats in the isogenic control?). To my knowledge, this line has not been published, even though it is commercially available from Cedars-Sinai. For example, see Coyne et al, Neuron, 2020 which used 20 iPSC cell lines, including one isogenic pair, but did not use this isogenic pair. Additional cell lines are critical given the new data suggesting that LSM12 and EPAC1 expression are reduced, suggesting for the first time in this paper, that these genes may play a role in C9 pathogenesis. If these findings were confirmed in multiple cell lines, this would greatly increase this reviewer's enthusiasm for publication.

In our revised manuscript, we examined multiple cell lines, including three C9-ALS iPSC lines (CS28, CS29, CS52) and three control iPSC lines (CS0, CS29-ISO, CS4), and confirmed that LSM12 and EPAC1 expression was consistently downregulated in all C9-ALS iPSC-derived neurons (Fig 7A and 7B). A previous study has shown that all these C9-ALS iPSC lines have mutant *C9ORF72* alleles with ~800 GGGGCC repeats (https://pubmed.ncbi.nlm.nih.gov/24154603/). Genomic deletion of the hexanucleotide repeat expansion in the isogenic control iPSC line of C9-ALS CS29 (CS29-ISO) has also been molecularly validated (https://pubmed.ncbi.nlm.nih.gov/32093728/). We cited these papers accordingly in the revised manuscript.

A second major concern I still have is the lack of evidence to support the title of the paper "LSM12-EPAC1 Defines a Neuroprotective Pathway that Sustains the Nucleocytoplasmic RAN Gradient". All three reviewers of the initial submission asked the authors for a relatively simple experiment to test their hypothesis: overexpress LSM12 and/or EPAC1 in their Drosophila model of C9-ALS, but the results of this experiment has not been described by the authors - presumably they were unable to perform the experiment or it gave the opposite results that they would have predicted. Instead, they overexpressed these genes in cell lines and show that it restores the Ran gradient and TDP-43 mislocalization. However, this is NOT sufficient evidence to claim that LSM12-EPAC1 are neuroprotective - this would require showing that

LSM12-EPAC1 rescue neuronal death or neurodegenerative phenotypes, neither of which are shown in this paper.

Our revised manuscript confirmed previous observations that C9-ALS iPSNs displayed baseline activation of the proapoptotic pathway, as assessed by high percentages of C9-ALS iPSNs expressing cleaved caspase-3 (https://pubmed.ncbi.nlm.nih.gov/27097283/, https://pubmed.ncbi.nlm.nih.gov/31019093/). We further showed that lentiviral overexpression of LSM12 or EPAC1 significantly suppressed the caspase-3 activation for apoptosis in all three C9-ALS iPSN lines (Fig 7G, 7H, and S12 Fig). We believe these new pieces of evidence address the reviewer's concerns above, better support our title, and make our revised manuscript suitable for publication in *PLOS Biology*.

Rev. 3:

The authors have submitted a substantially revised manuscript and most of my comments have been answered. The addition of human patient-derived neurons has much improved the manuscript and in its current form the manuscript is suitable for publication in PLos Biology. I have few minor comments, that would need to be addressed in the manuscript:

1) How long were the patient-derived neurons in culture and when were the experiments performed, DIV? would be good to include in the figure.

We performed gene expression analyses and transgenic experiments in control vs. C9-ALS iPSNs 21 days after neuronal differentiation from their neural progenitor cells. We clarified this in the revised manuscript.

2) What was the infection efficiency and were any survival measurements done?

Our recombinant lentiviruses were designed to express the indicated FLAG-tagged protein along with a GFP reporter, and we routinely found that ~70% of iPSNs were GFP-positive (Fig 7G). As an indirect readout for the survival measurements, we quantified cleaved caspase-3 signals in lentivirally infected iPSNs and found that overexpression of LSM12 or EPAC1 significantly suppressed the caspase-3 activation for apoptosis in all three C9-ALS iPSN lines.

3) Please provide some low magnification images showing large field of view for infection of neurons in Supplementary figure

We included low-magnification images for lentiviral infection of iPSNs in Fig 7G.

4) Was any Filter trap assays performed on patient-derived neurons to measure soluble versus insoluble Poly-GR aggregates

Our assessment of poly(GR) expression in patient-derived neurons was based on immunostaining (S10B Fig), and we did not have a chance to perform filter trap assays to measure soluble vs. insoluble poly(GR) aggregates in this study.

The manuscript has found a novel and interesting pathway that serves as a disease pathway and these findings are of interest to the field.
