# Supplementary material for: LSM12-EPAC1 defines a neuroprotective pathway that sustains the nucleocytoplasmic RAN gradient
Source: PLoS Biol. 2020 Dec 23;18(12):e3001002. doi: 10.1371/journal.pbio.3001002 (PMC7757817; doi:10.1371/journal.pbio.3001002)
Supplement: Attachment — Submitted filename: ResponseToReviewers_LeeLim2020PLOSBiol.docx [file pbio.3001002.s018.docx]

Please find our point-by-point responses below in blue.

Reviewers’ comments

Rev. 1:

In this study the authors look at the role of LSM12 in nucleocytoplasmic transport (NCT) and in maintaining the RAN gradient between the nucleus and the cytoplasm. They show it act via EPAC1 to maintain NCT and nuclear integrity. They go on to show that downregulation of these factors can worsen defects linked to ALS-associated toxic proteins both in cells and in flies. This paper is clear, well written and provides appropriately quantified data in support of their conclusions. The paper shows a novel role for LSM12 and EPAC1 in regulating NCT and the RAN gradient. Most of this is reasonably convincing (see minor comments below), however the disease relevance is less convincing as the authors do not show a rescue of disease-associated phenotypes. A rescue of GR associated toxicity, preferably in vivo in the flies (but also in cells) would increase disease relevance.

Major points:

1. A rescue of a GR toxicity phenotype is necessary to prove disease relevance. Either showing that over-expression of LSM12 or EPAC1 can rescue RAN gradient, nuclear integrity, NCT in GR over-expressing cells or show in the fly eye a rescue of the GR related neurodegeneration. There are stocks in Bloomington to over-express EPAC1 (stock 19033) and LSM12 (stock 15060). This would provide evidence that that these proteins are mediating the toxicity associated with GR over-expression, increasing the interest in the work.

We showed in the revised manuscript that overexpression of LSM12 or EPAC1 indeed rescued the RAN gradient and nuclear integrity in poly(GR)-overexpressing cells (please see Fig 6G-6J). We further employed iPSC-derived neurons from the *C9ORF72*-associated ALS patient (C9-ALS iPSNs) along with their CRISPR/Cas9-edited isogenic control iPSNs to demonstrate that 1) endogenous expression of *LSM12* and *EPAC1* was significantly down-regulated in C9-ALS iPSNs at both protein and transcript levels (Fig 7A and 7B); and that 2) lentiviral overexpression of LSM12 or EPAC1 rescued the RAN gradient and suppressed the pathogenic mislocalization of nuclear TDP-43 in the cytoplasm of C9-ALS iPSNs (Fig 7C-7F). By contrast, LSM12^V135I^ overexpression disrupted both of the RAN gradient and NCT even in isogenic control iPSNs (Fig 7C-7F). These new pieces of evidence convincingly support our conclusion that LSM12-EPAC1 defines a neuroprotective pathway for sustaining the RAN gradient, and validate its relevance to the pathophysiology of *C9ORF72*-associated ALS/FTD.

Minor points:

1. Fig 1C there is no quantification for the western blot even thought the authors are commenting on the levels of the proteins

We revised Fig 1C to show ATXN2 and LSM12 protein expression in different cell lines on the same blot. We further quantified their relative levels and averaged them from 3 independent experiments to statistically determine any significant difference in ATXN2 and LSM12 expression among those cell lines.

2. Fig 3C there appears to be a bleed-through of the red channel in the red (as the GR granules in red are appearing the green channel, will this interfere with the quantification?)

Some of the GR granule-positive cells displayed “bleed-through”-like signals in the green channel (i.e., S-GFP). Although they had little effects on the quantification, we excluded those cells from our analyses in the revised manuscript.

3. Fig 6A/B. To prove that EPAC1 and LSM12 are indeed acting in one linear pathway it would be nice to see an epistasis experiment showing that when both EPAC1 and LSM12 are knocked down the phenotype is not additive. The authors do a similar, nice experiment in Fig 1. As the experiments stand the defect from EPAC1 knock-down looks more severe, the authors should comment.

We indeed found that EPAC1 depletion did not further disrupt the RAN gradient in *LSM12*^KO^ cells (please see Fig S8B in the revised manuscript). The non-additive effects of EPAC1 depletion and *LSM12* deletion, as well as the rescue of *LSM12*-deletion phenotypes by EPAC1 overexpression (Fig 5E-5H), thus convincingly support that they act in a linear pathway for sustaining a RAN gradient. We think that EPAC1 depletion phenotypes are generally comparable to those seen in *LSM12*^shRNA^ or *LSM12*^KO^ cells. If the former were more severe than the latter, one possible explanation would be that endogenous EPAC1 levels in *LSM12*^shRNA^ or *LSM12*^KO^ cells might be higher than those in EPAC1-depleted cells.

4. Fig 6G the fly eye phenotype should be scored

The fly eye phenotypes were appropriately scored in the revised manuscript (please see Fig S9B).

Rev. 2:

This paper identifies a novel role for the Ataxin2-binding protein LSM12 in the post-transcriptional regulation of EPAC1, a protein previously shown to function at the nuclear pore complex through interactions with Ran and RanBP2. Unfortunately, though, this interesting finding is only briefly covered in one figure (Figure 5), and the paper instead attempts to connect this biology to the pathophysiology of ALS/FTD caused by mutations in the C9orf72 gene, for which their evidence is quite weak. There really is no evidence to support the major conclusion of the paper that LSM12-EPAC1 “defines a neuroprotective pathway” or that it functions in C9 ALS/FTD pathophysiology.

Major concerns:

1. Although many labs still use overexpression of polyPR or polyGR as a model of C9 ALS/FTD, increasing evidence casts doubt on the pathophysiologic relevance of overexpression of dipeptide repeats. PolyGR overexpression is extremely toxic to cells, and there is no evidence provided to suggest that they are studying an early event in this pathophysiologic cascade; for example, none of their manipulations of the LSM12/EPAC1 pathway rescue toxicity in cell or fly models. Since there are many cell culture and fly models that express the G4C2 hexanucleotide repeat expansion which is clearly upstream of polyGR production, these models should be used to validate findings observed in polyGR overexpression models. To show relevance to C9-ALS/FTD, ideally they would perform experiments in iPS neurons derived from C9-ALS/FTD patients.

In our revised manuscript, we employed C9-ALS iPSNs and showed that lentiviral overexpression of LSM12 or EPAC1 indeed rescued the RAN gradient and pathogenic mislocalization of nuclear TDP-43 in the cytoplasm of C9-ALS iPSNs (Fig 7C-7F). Please also see our response to a similar concern raised by the other reviewer (Reviewer #1, major point #1).

2. Their data quite clearly show that LSM12 regulates the Ran gradient independent of cell stressors. Even in the absence of oxidative stress or polyGR, LSM12 depletion leads to a dramatic alteration of the Ran gradient in cells (Fig 2C-D). Thus, I strongly disagree with their conclusion that their “data suggest that LSM12 establishes a RAN gradient that serves to sustain NCT under oxidative stress conditions” (top of p.5). LSM12 knockdown has a strong effect on the basal ran gradient in the absence of oxidative stress.

We agree that LSM12 and EPAC1 have a role in the establishment of a basal RAN gradient and that loss of their function may sensitize the affected cells to other cell stressors disrupting the RAN gradient and NCT (i.e., oxidative stress, poly(GR) overexpression, pathogenic repeat expansions in the *C9ORF72* locus). We accordingly clarified it in the revised manuscript.

3. Both polyGR and Epac1 deficiency independently disrupt nucleocytoplasmic transport, and there is very little evidence that these two findings are mechanistically related. For example, in Fig. 6A-B, both overexpression of polyGR and knockdown of EPAC1 lead to disruption of the Ran gradient, but there is no evidence that overexpression of EPAC1 can rescue the phenotype caused by polyGR expression in either cells or flies. To show a “neuroprotective” function of this pathway or relevance to C9-ALS/FTD, the authors need to show that upregulation of this pathway can rescue neurodegeneration.

The non-additive effects of poly(GR) and EPAC1 deficiency on the RAN gradient (Fig 6A and 6B), NCT (Fig S8C and S8D), and nuclear integrity (Fig 6E and 6F) indicate that they act on the same genetic pathway for these processes and thus are mechanistically related. If poly(GR) overexpression and EPAC1 depletion were not related, we should have just seen their additive effects. We further showed in the revised manuscript that overexpression of LSM12 or EPAC1 rescued the RAN gradient (Fig 6G and 6H) and nuclear integrity (Fig 6I and 6J) in poly(GR)-overexpressing cells. More importantly, the neuroprotective function of this pathway and its relevance to C9-ALS/FTD was validated by our new evidence obtained from C9-ALS iPSNs (Fig 7). Please also see our responses to similar concerns raised by the reviewers (Reviewer #1, major point #1; Reviewer #2, major concern #1).

4. No data is provided as to how the V135I variant is associated with ALS. Three GWAS studies are cited, but when I search the text of these papers, I do not find the LSM12 gene – presumably this variant was observed in a supplemental table in one of these studies, but observing this variant in an ALS cohort does NOT provide evidence of an association of the V135I allele with ALS. When I search Gnomad, this variant is present in the “apparently normal” population (two alleles), suggesting that if it is associated with ALS, it is likely a risk variant rather than causative.

We initially identified several LSM12 variants, including V135I, observed in ALS patients using the Project MinE data browser (http://databrowser.projectmine.com/). As the reviewer pointed out, this rare allele is also found in dbSNP (https://www.ncbi.nlm.nih.gov/snp/rs1162569843), but its low allele frequency does not allow us to determine whether or not this mutation is exclusively associated with ALS. We accordingly modified our text to clarify this issue. Please also see our response to the Reviewer #2, minor concern #2 below, regarding the risk vs. causative nature of LSM12 function in C9-ALS/FTD.

Minor concerns:

1. The method by which N/C ratio of S-GFP is calculated is not adequately described. In Figs 2D and 3D, the PBS controls are all 1.0, suggesting that all values are normalized to the control.

We modified our quantification method in the revised manuscript such that the N/C ratio values for the RAN gradient and NCT reporters were not normalized to the control.

2. Given the striking effect of LSM12 knockdown on the RAN gradient in PBS/DMSO condition (Figure 2C-D), what is the effect on NCT (S-GFP/tomato)? The Ran antibody appears to label signal at the cell cortex—is this background?

It is puzzling that depletion of LSM12 or EPAC1 disrupts the RAN gradient while not affecting NCT in the absence of cellular stress (i.e., arsenite-induced oxidative stress, poly(GR) overexpression). We speculate that there might be a compensating mechanism for LSM12 or EPAC1 deficiency in non-stressed cells to sustain NCT. However, this mechanism appears not to work properly under stress conditions, exaggerating the stress-induced NCT phenotypes in LSM12- or EPAC1-depleted cells. In this sense, LSM12 and EPAC1 likely act as a risk rather than a causative factor for NCT-relevant neurodegeneration, although overexpression of a specific *LSM12* allele (*LSM12*^V135I^) impaired both RAN gradient and TDP-43 transport in control iPSNs. Accordingly, we discussed it in the revised manuscript. Some microscopy settings during the acquisition of confocal images in the original Fig 2C likely have caused more anti-RAN signals at the cell cortex since we do not see a comparable pattern of the anti-RAN antibody staining in other figures. To avoid any unnecessary issues, we revised Fig 2C with new data.

3. What is “control” for LSM12 shRNA experiments (Figures 3 and 4)? There should be an shRNA control which does not appear to be the case.

We used an shRNA control that did not target any mammalian transcripts and clarified this in the revised manuscript.

4. That the LSM12[V135I] variant (but not WT) overexpression causes alteration of the Ran gradient is intriguing, but western analyses of LSM12 protein levels in these experiments needs to be done to ensure that the effect is not due to altered protein expression level. Similarly, there are no experiments to show that it is acting as a dominant-negative – to test that it is a dominant-negative vs a neomorphic change, they would need to see if coexpression of wildtype alleviates the phenotype.

Immunoblotting analyses revealed that LSM12^V135I^ overexpression did not alter endogenous levels of LSM12 protein in SH-S5Y5 cells (Fig S5). More importantly, LSM12^V135I^ overexpression negligibly affected the RAN gradient in *LSM12*-deleted cells while disrupting it in control cells (Fig 4F). On the other hand, LSM12^V135I^ overexpression impaired NCT even in the absence of oxidative stress, and these effects were observed in both control and *LSM12*^KO^ cells (Fig 4B). Thus, we concluded that this specific LSM12 variant displayed dominant-negative and neomorphic effects on the RAN gradient and NCT, respectively, and discussed it in the revised manuscript.

Rev. 3:

In this manuscript, the authors have identified that LSM12 together with EPAC1 or EPAC1 singularly confers neuroprotection via the suppression of the toxic Poly (GR) induced nucleocytoplasmic transport, which is observed in C9ORF72-associated ALS/FTD. In vitro assays performed on LSM12 depletion in human neuroblastoma cells intensified poly(GR)-induced impairment of nucleocytoplasmic transport, integrity and led to the formation of nuclear Poly(GR)granules, Further to provide a link to the disease-related pathology, they overexpressed a LSM12V135I mutation; rarely observed in ALS patients and observed enhanced poly (GR) toxicity, compromising nuclear integrity. Additionally, they show that LSM12 modulates EPAC1 expression, and EPAC1 reduction led to the dissociation of the RAN-importin β1 from the cytoplasmic nuclear pore complex, thereby perturbing the gradient required for efficient nucleocytoplasmic transport. In Drosophila, they provide evidence that EPAC1 mutations aggravate poly(GR)-induced neurodegeneration. Despite the fact that most experiments are performed using immortalized cell lines, and experiments are not always to the highest standards, overall, the findings are of interest to the ALS field.

1. One of the major concerns is that immortalized cell line is used, this reviewer can understand that primary cultures have some experimental limitations but some experiments should be confirmed in primary neurons or even in patient-derived pluripotent stem cells, as is the norm in the field. Indeed, SG assembly/disassembly is also influenced by the metabolic state as well as mitotic phases of cells.

Please see our responses to similar concerns raised by other reviewers (Reviewer #1, major point #1; Reviewer #2, major concern #1).

2. Similarly, only a single SG marker is employed to measure the appearance of SGs, other well-known markers need to be used to confirm their findings. Moreover, it is striking that they see mainly 1 or at times 2 SGs per cell in response to stress. One would imagine a sprinkling of SGs in a cell after stress. This reviewer found it hard to accept the claim “Further quantification revealed that each LSM12-depleted cell initially formed fewer SGs than control cells and displayed a smaller average size of SGs during maturation”. The images present in Fig 1a do not corroborate this statement. There are almost no cell within the panel 1A exhibiting 4-5 SGs within 30 or 60 min as shown in graphs in Fig 1B, D, E. How was the size of SGs measured in an unbiased manner? One would need 3D isosurface based analysis or ultrastructural analysis via EM. One wonders whether the stress paradigm is too mild. In this context, a non-chemical stress paradigm such as heat shock would be appropriately required to their findings.

In the revised manuscript, we employed anti-G3BP1 and anti-PABPC1 antibodies to assess *LSM12* effects on arsenite-induced SG formation (Fig 1 and S1 Fig) and revised Fig 1 with new data. The size of SGs was unbiasedly measured using ImageJ software as described previously (https://pubmed.ncbi.nlm.nih.gov/29523872/) and averaged from each confocal image. As the reviewer mentioned above, we applied mild/chronic (50 μM arsenite, ~4 hours) rather than acute (500 μM arsenite, 1 hour) oxidative stress to mimic physiologically relevant conditions and monitor the dynamics of SG assembly at a better temporal resolution. In the revised manuscript, we showed that LSM12-depleted cells still displayed loss-of-function phenotypes in arsenite-induced SG assembly, albeit weakly, under the acute oxidative stress conditions (please see Fig S1B and S1C). We also examined *LSM12* effects on SG formation under other stress paradigms (sorbitol/osmotic stress in Fig S2A; poly(GR) overexpression in Fig 3A and B), including the non-chemical stress, to see if LSM12-depletion phenotypes are specific to the arsenite-induced SG formation. We agree that 3D isosurface-based analysis or EM-based ultrastructural analysis would be more accurate for quantifying SG formation. However, *LSM12* effects on SG formation were readily detectable under these experimental conditions. One of the key findings in our manuscript is also the SG-independent function of the LSM12-EPAC1 pathway in the RAN gradient, NCT, and relevant pathologies in C9-ALS/FTD. Future studies should thus make use of those in-depth analyses of LSM12-dependent SG assembly and further elucidate the underlying mechanisms.

3. In the same line, no experiment was performed in the presence of puromycin; which promotes the formation SGs to better understand the role of LSM12 in slowing down the kinetics of SG formation. Further aspects of SG assembly versus disassembly have not been probed.

We found that puromycin promoted arsenite-induced SG formation in control^shRNA^ cells, but not in LSM12-depleted cells (please see Fig S1A in the revised manuscript). To further probe LSM12 effects on SG disassembly, we acutely induced SG formation with a high dose of arsenite and then traced SG disassembly after the removal of arsenite from cell culture media (please see Fig S1B and S1C in the revised manuscript). We observed the milder effects of LSM12 depletion on SG formation under acute oxidative stress. The number and size of SG became comparable between control and LSM12-depleted cells 3 hours after arsenite removal, and most SG disappeared in both cell lines 4 hours after recovery from arsenite treatment.

4. Authors have shown in drosophila overexpressing poly-GR, that reduction in EPAC1 levels worsens the phenotype, since poly-GR expression itself is sufficient to impair protein translation and stress granule dynamics (PMID: 29942091), reverse experiment needs to be performed where EPAC1 overexpression mitigates the poly-GR phenotype.

Please see our responses to similar concerns raised by other reviewers (Reviewer #1, major point #1; Reviewer #2, major concerns #1 and #3).

5. Are EPAC1 levels reduced in C9-ALS patient tissue or derived neurons? Some interrogation within a relevant disease model from patients is required.

We found that endogenous expression of *LSM12* and *EPAC1* is significantly down-regulated in C9-ALS iPSNs at both protein and transcript levels (please see Fig 7A and 7B in the revised manuscript). Please also see our responses to similar concerns raised by other reviewers (Reviewer #1, major point #1; Reviewer #2, major concern #1).

Immunoblot assays on protein lysates from C9-ALS patient-derived neurons demonstrated both LSM12 and EPAC1 protein levels were significantly reduced. Furthermore, we examined whether the decline in the LSM12 and EPAC1 protein level was reflected at the transcript level. We performed qRT-PCR and found LSM12 and EPAC1 transcript abundance significantly reduced in C9-ALS patient-derived neurons relative to controls, consistent with our SH-SY5Y data that both EPAC1 transcript and EPAC1 protein were expressed at low levels in either LSM12-depleted cells or LSM12-deleted cells.

Minor comments

6. Figure 1C requires the experiment to be shown on the same blot.

We revised Fig 1C to show ATXN2 and LSM12 protein expression in different cell lines on the same blot. Please also see our response to Reviewer #1, minor point #1.

7. Labeling of X-axis in graphs (1B, D and E) is missing minutes. Fig 3E, has a B on the image in the lower panel.

We corrected them in the revised manuscript.

8. Discussion needs to be modified and key physiological role of C9orf72 in SGs assembly/disassembly needs to be included (see references: PMID: 27037575, PMID: 30022074), rather than limiting the discussion to Poly-GR, which is only one out of the five toxic DPRs in C9-ALS-FTD context.

We discussed the physiological role of *C9ORF72* in SG assembly/disassembly and cited those references in the Introduction as we feel it fits better into that context.

9. There is no indication of how many times each experiment was repeated, often there is huge variation range in the number of cells analyzed, for e.g. (n = 10 confocal images; n = 90–453 cells). For each condition the number of times the experiment was performed, the number of images and cells quantified should be clearly stated.

In the revised manuscript, we clearly stated the number of independent experiments performed and the number of confocal images and cells quantified in each figure legend. The efficiency of our transient transfection was not 100%. Also, we generally observed lower numbers of GFP-GR_100_-expressing cells than GFP-expressing cells, leading to variations in the number of green fluorescence-positive cells we could analyze from each confocal image.
