# Supplementary material for: LSM12-EPAC1 defines a neuroprotective pathway that sustains the nucleocytoplasmic RAN gradient
Source: PLoS Biol. 2020 Dec 23;18(12):e3001002. doi: 10.1371/journal.pbio.3001002 (PMC7757817; doi:10.1371/journal.pbio.3001002)
Supplement: Attachment — Submitted filename: Response To PBIOLOGY-D-20-01607 PLOS Formatting Requests.docx [file pbio.3001002.s021.docx]

PLOS Formatting Requests

# For: “LSM12-EPAC1 Defines a Neuroprotective Pathway that Sustains the Nucleocytoplasmic RAN Gradient”

To access your manuscript and complete these changes, please follow the link in the email and find your manuscript in the ‘Submissions Needing Revision’ folder on your main menu. Please also provide a tracked changes file indicating any updates.

**This is your FINAL opportunity to make amendments to the scientific content of your manuscript.** We strongly recommend that all authors double check ALL files prior to the final versions being submitted. Content changes requested at later stages WILL NOT BE ACCEPTED.

**Questions?** Email [plosbiology@plos.org](mailto:plosbiology@plos.org) quoting your manuscript number. We also recommend reading our [author submission guidelines.](http://journals.plos.org/plosbiology/s/submission-guidelines)

# METADATA CHECK

Please ensure that all information entered in the submission form is correct. This information will be indexed in PubMed and published by *PLOS Biology* as written.

- An **Early Version** of your manuscript:
  - WILL NOT be published online ahead of the final version

Confirmed.

If you (or your institution) are planning a press release for this article, please contact us as soon as possible to ensure that an early version of your paper is not published.

- Please check the **authorship and affiliations** list on the title page of your manuscript. These details will be used to index the authorship of your manuscript and therefore should be checked carefully for any errors.

Confirmed.

- Please ensure that if you have any protocols associated with [protocols.io](https://www.protocols.io/) in your manuscript, they are made public before publication to guarantee immediate access to the methodological details.

N/A.

Please confirm that the following elements are correct and written in full sentences. Please update as needed.

- **Financial Disclosure**
- **Competing Interests**
- **Data Availability Statement**

All confirmed.

# MANUSCRIPT REQUIREMENTS

Please update your manuscript with the following changes:

1. Please complete the Blurb section of the submission form. This will be included in our weekly and monthly Electronic Table of Contents, sent out to users signed up to receive alerts from PLOS Biology. The blurb should be about 30-40 words long and is subject to editorial changes. It should, without exaggeration, entice people to read your manuscript. It should not be redundant with the title and should not contain acronyms or abbreviations. For examples, please see our Author Guidelines: <http://journals.plos.org/plosbiology/s/revising-your-manuscript>.

We have completed the Blurb section of the submission form: “A post-transcriptional circuit of LSM12-EPAC1 suppresses neurodegenerative pathologies in C9ORF72-associated amyotrophic lateral sclerosis by establishing the RAN gradient and sustaining nucleocytoplasmic transport.”

2. In your Data Availability Statement, please ensure you have provided all necessary access information in complete sentences. This statement will appear on the published version of your manuscript.

We have provided all necessary accession information in our Data Availability Statement: “All relevant data are within the paper and its Supporting Information files. Raw and processed data from RNA sequencing experiments can be downloaded from GEO (accession number GSE160159).”

3. Please ensure that all authors have at least one affiliation listed on the title page and that all listed affiliations are attributed to at least one author. Affiliations should be listed in ascending numerical order in the affiliation list and should match what is provided in the Authors Information in the submission form.

   Affiliations should include an institution, a city, and a country (no postcode). US affiliations need the state as well, written out in full (with no abbreviations).

Confirmed and corrected.

4. When referring to your Materials and Methods/Models/Methods section throughout the text, please use the full title rather than “Methods”, or change the title of the section to match the references in the text.

The full title was used in the revised manuscript.

5. We have noted that you have the following present in your manuscript: Financial Disclosure (Funding)/ Competing Interests statement/ Author Contributions

We will remove these sections from your manuscript file as this information is automatically pulled from the submission form. Please make sure that the information in the submission form is correct.

Funding and Author contributions were removed from the revised manuscript.

6. You currently have references stating '[data] not shown' on page 30. Please note that PLOS does not permit references to “data not shown.” Authors should provide the relevant data within the manuscript, the Supporting Information files, or in a public repository. If the data are not a core part of the research study being presented, we ask that authors remove any references to these data.

As the data were not a core part of the research study being presented, we removed the reference to them in the revised manuscript.

7. Please make sure all references are cited in ascending numerical order in the text.

Confirmed.

8. Please upload your Supporting Information items individually, rather than in a single file, as you refer to them individually in the text. If you would like to retain the single file please rename internal items (e.g. tables) as A, B, C etc. Please also provide a legend for each under the heading Supporting Information. Supporting Information files should be less than 10 MB each. Please edit any citation of internal items within your Supporting Information files to using letters rather than numbers, e.g. “Table A in S1 Text”. Please ensure these changes are made throughout the manuscript and within the Supporting Information files themselves.

Confirmed.

9. Please ensure that all Supporting Information files use the following format verbatim throughout the manuscript (including legends and all in-text citations): S1 Fig, S1 Table, S1 Text, S1 Data, etc. Each file type should start at 1 and increase numerically. Please note, however, that the file names themselves must use an underscore rather than a space, i.e. S1_Fig.tif, S1_Table.xlsx, S1_Text.doc, S1_Data.xlsx, S1_PRISMA_Checklist.docx, etc.

Confirmed.

10. Please ensure that each supporting information file has a legend at the end of the manuscript file, after the Reference list.

Confirmed.

11. Please note that your submission cannot contain any references to generic "supporting information" files and instead must refer to specific files using our naming convention (S1 Fig, S1 Table, S1 Text, S1 Data, etc.)

Confirmed.

**Before resubmitting your paper, please ensure that all files referenced in the manuscript are present in the File Inventory, as missing files will result in further delays. Please also ensure that all file names adhere to our naming conventions and that all in-text citations and supporting information file legends/captions do as well.**

If Supporting Information files are included with your article, note that these are not copyedited and will be published as they are submitted. Please ensure that these files are legible and of high quality (at least 300 dpi) in an easily accessible file format. For this reason, please be aware that any references listed in an SI file will not be indexed. For more information, see our [Supporting Information guidelines](http://journals.plos.org/plosbiology/s/supporting-information).

# FIGURE REQUIREMENTS

PLOS offers a figure-checking tool, PACE (<http://pacev2.apexcovantage.com/>), to help authors to ensure all figures meet PLOS requirements so that the quality of published figures is as high as possible. **Please use this tool to help you address the requests below.**

PACE is a digital diagnostic and conversion tool for figure files. It will provide information about any failed check(s) and, if able, will automatically convert the figure file into an acceptable file that passes quality checks. PACE requires you to register for an account to ensure your figure files are processed securely.

Please complete the following instructions regarding your figure files:

1. Please ensure that all main figures are referenced in the manuscript as Fig 1, Fig 2, etc. (including capitalization), rather than Figure 1, Figure 2, etc. Please note, however, that the file names themselves must not include the space, i.e. Fig1.tif, Fig2.tif. Please ensure that all main figure files are clearly labeled as such in the File Inventory.

Confirmed.

2. Please confirm that you either created the following figure panels, or that you will replace them with an open source alternative: graphics/illustrations in S15 Fig

We created graphics/illustrations in S15 Fig.

Here are a few open source image websites you may wish to use:
- <https://commons.wikimedia.org>
- <http://www.publicdomainpictures.net/>
- <https://openclipart.org/>

Please note that this is your only chance to adjust figures, changes to figures (including resizing) at proof will not be accepted.

For a full outline, please see our [figure](http://journals.plos.org/plosbiology/s/figures) and [table](http://journals.plos.org/plosbiology/s/tables) preparation guidelines.

You can also upload a Striking Image to accompany your article if one is available. If this image is judged to be suitable, it may be featured on our website. Images should be high resolution, eye-catching, single panel images. If your image is from someone other than yourself, please ensure that the artist has read and agreed to the terms and conditions of the Creative Commons Attribution License. Note: we cannot publish copyrighted images.
